# PD-L1 knockdown suppresses vasculogenic mimicry of non-small cell lung cancer by modulating ZEB1-triggered EMT

**DOI:** 10.1186/s12885-024-12390-8

**Published:** 2024-05-23

**Authors:** Wenjuan Li, Jiatao Wu, Qianhao Jia, Yuqi Shi, Fan Li, Linxiang Zhang, Fan Shi, Xiaojing Wang, Shiwu Wu

**Affiliations:** 1 Anhui Province Key Laboratory of Clinical and Preclinical Research in Respiratory Disease, Molecular Diagnosis Center, First Affiliated Hospital, Bengbu Medical University, 287 Changhuai Road, Bengbu, Anhui 233004 China; 2 Department of Pathology, The First Affiliated Hospital of Bengbu Medical University, Bengbu, People’s Republic of China; 3 Department of Pathology, Bengbu Medical University, Bengbu, 233030 Anhui China; 4 Department of Pathology, Anhui No.2 Provincial People’s Hospital, Hefei, China

**Keywords:** PD-L1, Vasculogenic mimicry, ZEB1, Non-small cell lung cancer, Epithelial-to-mesenchymal transition

## Abstract

**Background:**

PD-L1 overexpression is commonly observed in various malignancies and is strongly correlated with poor prognoses for cancer patients. Moreover, PD-L1 has been shown to play a significant role in promoting angiogenesis and epithelial-mesenchymal transition (EMT) processes across different cancer types.

**Methods:**

The relationship between PD-L1 and vasculogenic mimicry as well as epithelial-mesenchymal transition (EMT) was explored by bioinformatics approach and immunohistochemistry. The functions of PD-L1 in regulating the expression of ZEB1 and the EMT process were assessed by Western blotting and q-PCR assays. The impact of PD-L1 on the migratory and proliferative capabilities of A549 and H1299 cells was evaluated through wound healing, cell invasion, and CCK8 assays following siRNA-mediated PD-L1 knockdown. Tube formation assay was utilized to evaluate the presence of VM structures.

**Results:**

In this study, increased PD-L1 expression was observed in A549 and H1299 cells compared to normal lung epithelial cells. Immunohistochemical analysis revealed a higher prevalence of VM structures in the PD-L1-positive group compared to the PD-L1-negative group. Additionally, high PD-L1 expression was also found to be significantly associated with advanced TNM stage and increased metastasis. Following PD-L1 knockdown, NSCLC cells exhibited a notable reduction in their ability to form tube-like structures. Moreover, the levels of key EMT and VM-related markers, including N-cadherin, MMP9, VE-cadherin, and VEGFA, were significantly decreased, while E-cadherin expression was upregulated. In addition, the migration and proliferation capacities of both cell lines were significantly inhibited after PD-L1 or ZEB1 knockdown.

**Conclusions:**

Knockdown PD-L1 can inhibit ZEB1-mediated EMT, thereby hindering the formation of VM in NSCLC.

**Supplementary Information:**

The online version contains supplementary material available at 10.1186/s12885-024-12390-8.

## Background

Lung cancer, a global disease with the second-highest incidence and highest mortality rate, exhibits an annual increase. Its incidence is also escalating in China. Moreover, it is the primary cause contributing to cancer-related mortality globally [1–6]. The symptoms of non-small cell lung cancer (NSCLC), accounting for approximately 85% of cases, tend to be subtle in the initial stages, resulting in a higher prevalence of diagnoses at middle and advanced stages. Despite advancements in treatment, the cure rate of NSCLC patients has not elevated significantly, recurrence and metastasis are still the major reasons for this [7, 8]. Tumor invasion and metastasis are closely associated with angiogenesis. Besides, tumor angiogenesis is considered to be one of the urgent issues in the field of oncology. However, antivascular therapy has not shown a marked improvement in survival rates for lung cancer patients and may even foster the development of vasculogenic mimicry (VM) [9]. Therefore, VM is believed to play a significant role in the progression of NSCLC and could be a key factor contributing to the inefficacy of anti-angiogenic therapies.

In 1999, Maniotis et al. introduced the concept of vasculogenic mimicry (VM) [10] to describe a characteristic phenomenon observed in aggressive melanoma cells. This phenomenon involves the production of a lumen-like structure independent of vascular endothelial cells, in which red blood cells are visible. The structure can communicate with the host’s microcirculatory system, thereby promoting tumor perfusion [11–14]. VM promotes tumor growth, and its direct communication with the host’s blood vessels often leads to metastasis and spread to other organs through the bloodstream, ultimately resulting in poor prognosis [15–18]. It has been demonstrated that VM is observed in various malignancies, including gastric, breast, and colon cancer, among others [19–22]. The association between VM and poor prognosis suggests that targeting VM could hold potential for anti-cancer therapy.

Programmed death protein ligand-1 (PD-L1), also known as B7-h1 or CD274, belongs to the B7/CD28 family and is an immunomodulatory ligand. PD-L1 plays a crucial role in regulating human immune function by binding to the programmed death protein 1 (PD-1) receptor on the T cell’s external membrane. This interaction results in immune cells losing their ability to attack, thereby protecting normal tissues from undesired attacks [23, 24]. Tumor cells exploit this mechanism by inducing increased expression of PD-L1 on their surface, enabling them to evade host immune cell attacks and ultimately leading to immune escape [25, 26]. Numerous studies have demonstrated that PD-L1 is widely expressed in various types of cancers and is strongly associated with poor prognosis [27–29]. Furthermore, the over-expression of PD-L1 has been closely linked to the EMT phenotype [28, 30, 31], which can promote tumor tissues angiogenesis [32]. However, it remains unclear whether heightened PD-L1 expression can promote the formation of vasculogenic mimicry in NSCLC.

Epithelial-mesenchymal transition (EMT) is a complex biological process observed in malignancies, involving the disruption of intercellular polarity and cohesive adhesion in epithelial cancer cells. This results in their transformation into single, nonpolarized, motile, and invasive mesenchymal cells [33, 34]. Actin cytoskeleton remodeling and significant alterations in gene expression, including transcription factors and related genes, are key components of this transition [35]. As one of the important regulators of EMT, ZEB1 exerts its influence by inhibiting epithelial genes while activating mesenchymal gene transcription through its binding to the initiation sequence of epithelial genes, ultimately facilitating the occurrence of EMT [36–38]. Numerous studies have provided substantial evidence demonstrating that the promotion of VM structure formation in malignancies is significantly attributed to the pivotal role of EMT, which enhances cell migration and proliferation [18, 22, 39–43]. .

This study aimed to investigate the function of PD-L1 in the progression of vasculogenic mimicry in NSCLC. To address this objective, we utilized siRNA to reduce the overall expression of PD-L1. Subsequently, we observed its impact on VM formation and epithelial-mesenchymal transition markers. The primary objective of this study was to identify whether PD-L1 holds the potential for anti-vascular therapy in NSCLC.

## Methods

### Clinical specimens and immunohistochemistry

A cohort of 100 patients diagnosed with NSCLC at the First Affiliated Hospital of Bengbu Medical University contributed postoperative tissue samples, which were collected prospectively. Patient selection for this study was conducted through randomization, ensuring that none of the participants had received radiotherapy or chemotherapy prior to surgery. The specimens were fixed, embedded, and subsequently subjected to tissue section staining to confirm the pathological diagnosis. The clinicopathological stage of the cancer was assessed based on the American Joint Committee on Cancer 8th edition cancer staging system. Detailed clinicopathological parameters of the patients can be found in Table 1.

**Table 1:** Clinicopathological characteristics of NSCLC

Patients characteristics	Frequency (*n*)	Percentage (%)
Age		
<60 years	38	38
≥60 years	62	62
Sex		
Male	31	31
Female	69	69
Smoking		
No	63	63
Yes	37	37
Tumor size (cm)		
≤3	44	44
>3	56	56
Gross Type		
Central	52	52
Peripheral	48	48
Histological Type		
SCC	41	41
Ade	59	59
Grade		
Well	22	22
Moderate	51	51
Poor	27	27
LNM		
No	53	53
Yes	47	47
TNM stage		
I	41	41
II	28	28
III+IV	31	31

The procedures of immunohistochemistry experimental and criteria for scoring the results were conducted following the methods outlined in a previous paper [44]. Two senior pathologists were then assigned to independently score the immunohistochemical results in accordance with the aforementioned criteria. Double staining with PAS-CD31 was then conducted to ascertain the presence or absence of vasculogenic mimicry (VM) structures. Tumor samples were identified as containing VM structures when they exhibited negative CD31 staining and positive PAS staining.

### Cell lines and culture

The human non-small cell lung cancer (NSCLC) cell lines A549, H1299, and PC9, as well as the normal lung epithelial cell line BEAS-2B, were acquired from the American Type Culture Collection (ATCC). The BEAS-2B cell line was cultured in a basal medium supplemented with 10% fetal bovine serum. In contrast, the A549, H1299, and PC9 cells were sustained in the complete PRMI-1640 medium, which was selected as a standard medium based on its wide utilization and demonstrated ability to support the growth and maintenance of these cell lines. All cell lines were incubated in a controlled environment with 37℃ and 5% carbon dioxide concentration.

### SiRNA transfection assay

Small interfering RNA (siRNA) targeting PD-L1 knockdown was transfected using LIPO8000 (C0533; Beyotime; China) according to the manufacturer’s instructions. In a six-well plate, for instance, 100 pmol of siRNA and 4 µl of LIPO8000 reagents were sequentially added to 125 µl of basal DMEM to achieve a homogeneous mixture, which was then incubated at room temperature for 20 min. After removing the previous medium from the cells to be transfected, the fresh medium was added to provide optimal conditions for subsequent steps. Following a 20-minute incubation period, the transfection mixture was added drop by drop to the cells. Western blotting was employed to detect and quantify PD-L1 protein, while q-PCR provides a sensitive and accurate measurement of PD-L1 mRNA expression.

### Lentiviral transduction

Control lentivirus and shRNA encoding for targeting ZEB1 were purchased from Shanghai He Yuan Biotechnology Company. Transduction was performed following the lentiviral transduction protocol. The A549 and H1299 cell lines were transduced with lentivirus and co-transducers (Heyuan; Shanghai; China). Then screened with puromycin for two weeks. Finally, assessed the ZEB1 knockdown efficiency and select the one with the highest efficiency for subsequent experiments.

### Western blotting analysis

The protein of the cells was extracted and quantified with RIPA buffer. It was isolated on SDS-PAGE and transferred to the PVDF membrane (Millipore, Billerica, MA, USA). After blocking with 5% skimmed milk powder for about 90 min, the membrane was washed using TBST on a shaker for 5 min each time, for a total of three times. Lastly, the PVDF membrane was incubated in a shaker at 4 °C for about 16 h with the corresponding primary antibody which had been diluted in a certain proportion such as rabbit anti-PD-L1 (1:700; 17952-1-AP; Proteintech), rabbit anti-ZEB1 (1:1500; bs-21785R; Bioss), rabbit anti-N-cadherin (1:750; BST17153912; Boster), rabbit anti-E-cadherin (1:1000; bs-1519R; Bioss), rabbit anti-VE-cadherin (1:1000; AF6265; Affinity), rabbit anti-MMP9 (1:1500; AF5228; Affinity), rabbit anti-VEGFA (1:1000; 26157-1AP; Proteintech), mouse anti-MMP2 (1:2000; 66366-1-Ig; Protientech), rabbit anti-GAPDH (1:20000; AB0037; Abways). The PVDF membrane, upon completion of protein transfer, underwent a thorough washing process on the following day to ensure the removal of unbound substances and optimize the signal-to-noise ratio. Then washed again after incubation with the secondary antibody as goat anti-rabbit (1:10,000; BL003A; Biosharp) and anti-mouse IgG (1:10,000; BL001A; Biosharp) for 1.5 h in the shaker. Finally, detected all the membranes after dropping ECL (Beyotime; China) and photographed using a developing instrument, with greyscale values calculated by Image J software and statistical analysis by GraphPad Prism 9.0. (To facilitate antibody incubation targeting proteins from different regions, we trimmed the PVDF membrane before the primary antibody incubation.)

### Reverse transcription quantitative real-time polymerase chain reaction (RT-qPCR)

Total RNA was extracted from A549 and H1299 cells using FastPure Cell/Tissue Total RNA Isolation Kit V2(Vazyme Biotech; Nanjing; China). Following RNA extraction, cDNA synthesis was carried out with the NovoScript®Plus All-in-one 1st Strand cDNA Synthesis SuperMix (gDNA Purge) (NOVO protein, Jiangsu, China). RT-qPCR analysis was performed using the NovoStart®SYBR qPCR SuperMix Plus (NOVO protein, Jiangsu, China). The human β-Actin gene was used as an internal reference gene, and the relative expression of mRNA was calculated using the 2^(-ΔΔCt) method. Statistical analysis by GraphPad Prism 9.0 software. Primers were designed using Primer 5. 0 (Table 2).

**Table 2:** Primer used in quantitative real-time PCR assays

Primer	Sequences(5′→ 3′)
ACTIN-F	GCCAACACAGTGCTGTCTGG
ACTIN-R	CTAAGTCATAGTCCGCCTAGAAGCA
PD-L1-F	TTTCAATGTGACCAGCAC
PD-L1-R	GGCATAATAAGATGGCTC
ZEB1-F	GTGACGCAGTCTGGGTGTAA
ZEB1-R	TGAGTCCTGTTCTTGGTCGC
E-cadherin-F	GCTGGACCGAGAGAGTTTCC
E-cadherin-R	CAAAATCCAAGCCCGTGGTG
N-cadherin-F	AATCGTGTCTCAGGCTCCAA
N-cadherin-R	TGGGATTGCCTTCCATGTCT
VE-cadherin-F	GTCGATGCAGAGACAGGAGA
VE-cadherin-R	GGTGAAGCTGGAAGGAGTCT
MMP9-F	GGGACGCAGACATCGTCATC
MMP9-R	TCGTCATCGTCGAAATGGGC
MMP2-F	GATACCCCTTTGACGGTAAGGA
MMP2-R	CCTTCTCCCAAGGTCCATAGC
VEGFA-F	AGGAGGAGGGCAGAATCATCA
VEGFA-R	CTCGATTGGATGGCAGTAGCT

**Table 3:** PD-L1 expression and clinicopathologic characteristics in the 100 non-small cell lung cancers

Factors	PD-L1 negative group (*n* = 28)	PD-L1 positive group (*n* = 72)	*p* value
Age			
<60 years (*n* = 38)	11 (39%)	27 (38%)	ns
≥60years (*n* = 62)	17 (61%)	45 (62%)	
Sex			
Male (*n* = 69)	17 (61%)	52 (72%)	ns
Female (*n* = 31)	11 (39%)	20 (28%)	
Smoking			
No (*n* = 63)	21 (75%)	42 (58%)	ns
Yes (*n* = 37)	7 (25%)	30 (42%)	
Tumor size (cm)			
≤3 (*n* = 44)	20 (71%)	24 (33%)	0.002
>3 (*n* = 56)	8 (29%)	48 (67%)	
Gross Type			
Central (*n* = 52)	12 (43%)	40 (56%)	ns
Peripheral (*n* = 48)	16 (57%)	32 (44%)	
Histological Type			
SCC (*n* = 41)	8 (29%)	33 (46%)	ns
Ade (*n* = 59)	20 (71%)	39 (54%)	
Grade			
Well (*n* = 22)	6 (21%)	16 (22%)	ns
Moderate (*n* = 51)	16 (57%)	35 (49%)	
Poor (*n* = 27)	6 (21%)	21 (29%)	
LNM			
No (*n* = 53)	23 (82%)	30 (42%)	0.001
Yes (*n* = 47)	5 (18%)	42 (58%)	
TNM stage			
I (*n* = 41)	18 (64%)	23 (32%)	0.004
II (*n* = 28)	9 (32%)	19 (26%)	
III+IV (*n* = 31)	1 (4%)	30 (42%)	

ns, not significant.

**Table 4:** Vasculogenic mimicry presence and clinicopathologic characteristics in the 100 non-small cell lung cancers

Factors	VM negative group (*n* = 64)	VM positive group (*n* = 36)	*p* value
Age			
<60 years (*n* = 38)	26 (41%)	12 (33%)	ns
≥60years (*n* = 62)	38 (59%)	24 (67%)	
Sex			
Male (*n* = 69)	42 (66%)	27 (75%)	ns
Female (*n* = 31)	22 (34%)	9 (25%)	
Smoking			
No (*n* = 63)	41 (64%)	22 (61%)	ns
Yes (*n* = 37)	23 (36%)	14 (39%)	
Tumor size (cm)			
≤3 (*n* = 44)	24 (38%)	20 (56%)	ns
>3 (*n* = 56)	40 (63%)	16 (44%)	
Gross Type			
Central (*n* = 52)	33 (52%)	19 (53%)	ns
Peripheral (*n* = 48)	31 (48%)	17 (47%)	
Histological Type			
SCC (*n* = 41)	28 (44%)	13 (36%)	ns
Ade (*n* = 59)	36 (56%)	23 (64%)	
Grade			
Well (*n* = 22)	12 (19%)	10 (28%)	ns
Moderate (*n* = 51)	36 (56%)	15 (42%)	
Poor (*n* = 27)	16 (25%)	11 (31%)	
LNM			
No (*n* = 64)	44 (69%)	9 (25%)	<0.001
Yes (*n* = 36)	20 (31%)	27 (75%)	
TNM stage			
I (*n* = 41)	34 (53%)	7 (19%)	0.004
II (*n* = 28)	15 (23%)	13 (36%)	
III+IV (*n* = 31)	15 (23%)	16 (44%)	

ns, not significant.

### Cell invasion assay

The Matrigengel Matrix (ABW; Shanghai; China) was diluted at a ratio of 1:8 with the basal culture medium PRMI-1640 and coated with the transwell chamber at 55ul per chamber. Following this, it was then coagulated in the incubator for 1 h. The desired cells were slightly detached, counted, and resuspended in basial PRMI-1640 medium. Subsequently, a concentration of 1 × 10^5^ cells was introduced into the upper chamber, while complete PRMI-1640 medium was added to the lower chamber to provide the necessary driving force for the cells. The system was then maintained at 37 °C, 5% CO_2_ for 24 h. Wiped off the upper chamber cells with a cotton swab, and washed it gently. The transwell chamber was placed in a 4% paraformaldehyde solution and allowed to incubate for approximately 15 min. It was washed subsequently as described earlier. Then the transwell inserts were immersed in a crystal violet staining solution and incubated for approximately 20 min to enable staining of migrating cells. The transwell inserts were then washed with PBS more than three times to remove any unbound staining solution. Finally, the transwell inserts were observed and photographed under a microscope. Image J software was used for analysis, and GraphPad Prism 9 was employed for data processing.

### Cell migration assay

The experimental procedures were carried out using the same protocol as the cell invasion assay mentioned earlier, with the exception of not coating the wells with matrix gel.

### Bioinformatic analysis

We analyzed the association between PD-L1 and ZEB1 in LUAD and LUSC using the GEPIA online dataset. Additionally, we applied the same methodology to investigate the correlation of PD-L1 with other relevant genes. The gene expression profiles and associated characteristics of the samples were obtained from the TCGA dataset. Additionally, Gene Set Enrichment Analysis was performed to analyze the relationships among them.

### Wound-healing assay

The cells were enzymatically dissociated, counted, and then resuspended before being seeded into six-well plates at a concentration of 1 × 10^6^ cells per well. Following seeding, cultured the cells for a duration of 24 h. When the cells reached 80-90% confluency, scratches were made using 20ul tips. The cells belonging to the same treatment group did not require tip changes. PBS gently washed three times and removed the shedding cells. Changed to low serum medium (1% FBS) for culturing. Micrograph images of the samples were captured at 0, 24, and 48 h using a microscope, respectively. Image J software was used for Calculating the cellular mobility, and the data were processed using GraphPad Prism 9.0.

### Three-dimensional tube formation assay

The matrix gel was thawed in advance, and the required tips and 96-well plate were pre-cooled. Each well was supplemented with 55 µL of matrix gel, followed by incubation at 37 °C for one hour to induce gelation. The cells were digested using trypsin, collected as a cell suspension, and subsequently counted. Slowly dropped the cell suspension onto Matrigel at 5 × 10^4^ per well. Images of the tube formation were captured at 16 h using a microscope. Image J software was used for calculating the number of tubes, and the data were processed using GraphPad Prism 9.0.

### Cell viability assay

Cells were resuspended after digesting and counting in PRIM-1640 as mentioned above. Then the cells were inoculated into 96 well cell culture plates, with each well containing a concentration of 2000 cells. At 24 h, 48 h, 72 h, and 96 h, the medium in each well was replaced with fresh medium. A volume of 10 µL of CCK8 reagent (C0039; Beyotime, China) was then added to it. The samples were subsequently placed in an incubator and incubated at 37 °C for 2 h. GraphPad Prism 9.0 was employed for data processing.

### Cell apoptosis assay

The cell culture medium was transferred into a suitable centrifuge tube, the cells were washed once with pre-chilled PBS. Then an appropriate amount of cell digestion solution containing trypsin was added for cell digestion. It was incubated at room temperature until gentle tapping allowed for cell detachment. Subsequently, the collected cell culture medium from the previous step was added, the cells were gently dislodged and transferred to the centrifuge tube. The centrifugation was performed at 1000 g for 5 minutes, the supernatant was discarded, and the cells were collected. The cells were gently resuspended in PBS and a cell count was performed. 1 × 10^5^ resuspended cells were transferred to a 1.5 ml Eppendorf tube, centrifuged at 1000 g for 5 minutes, and the supernatant was discarded. Then, 195 µl of Annexin V-FITC binding solution, 5 µl of Annexin V-FITC, and 10 µl of propidium iodide staining solution (C1062; Beyotime, China) were sequentially added, with gentle mixing after each step. Finally, it was incubated in the dark at room temperature for 20 min with aluminum foil. Followed by incubation in an ice bath for subsequent flow cytometry analysis. The cells were resuspended 2–3 times during the incubation process to enhance staining efficiency. Data processing was performed using GraphPad Prism 9.0.

### Statistical analysis

Statistical analysis of the data was conducted using GraphPad Prism 9.0 software. The mean ± standard deviation was presented for results obtained from three or more independent experiments. To compare two groups, paired or two independent samples t-tests were conducted, while the chi-square test was utilized for comparing count data. For comparisons between multiple groups, one-way ANOVA was employed. To assess correlations between two variables, linear regression analysis was conducted. Statistical significance was defined as p-values < 0.05.(*, p value<0.05; **, p value<0.01; ***, p value<0.001; ****, p value<0.0001.)

## Results

### Upregulation of PD-L1 in NSCLC and its correlation with unfavorable prognosis in patients

**Fig. 1:**
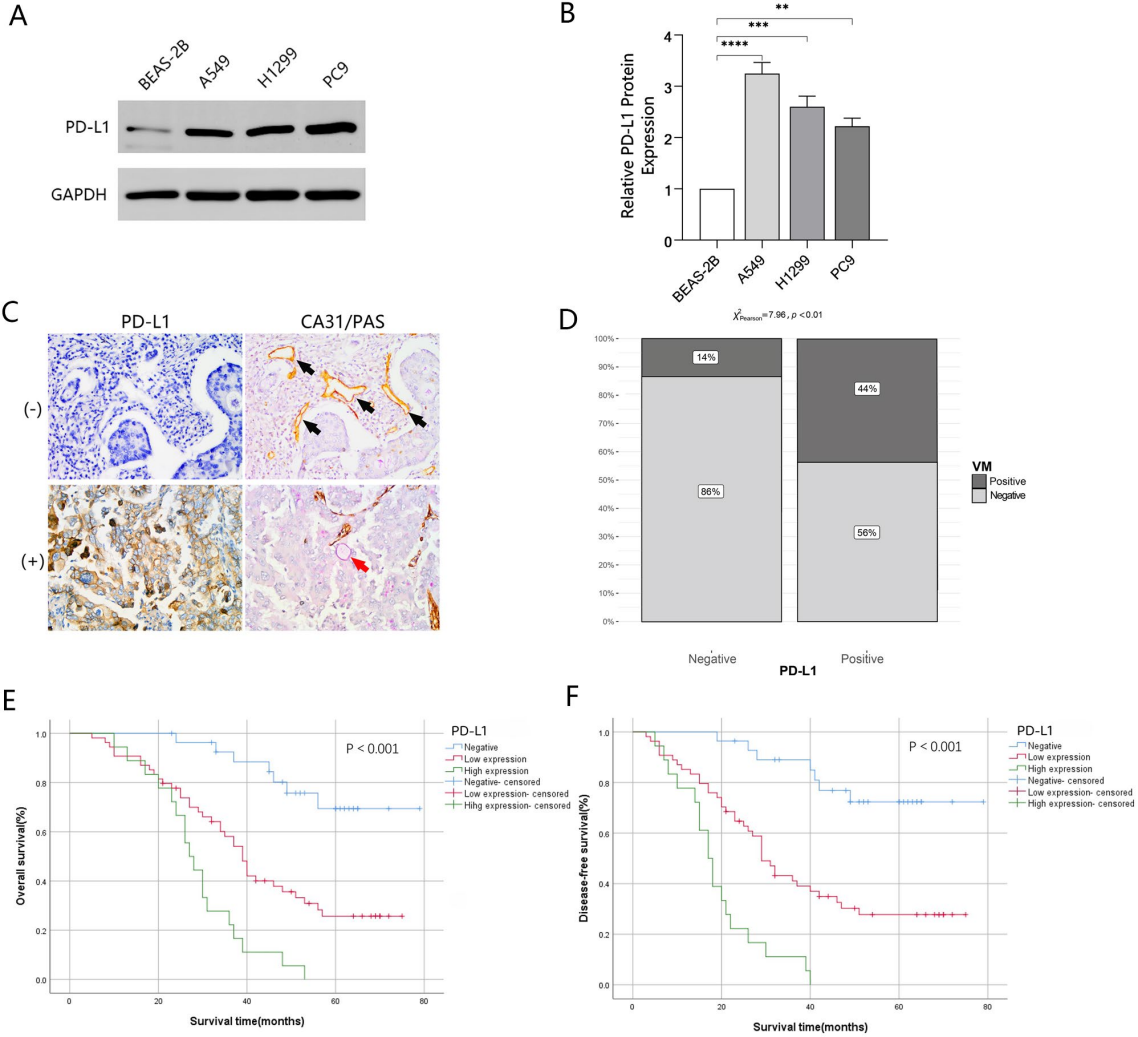
PD-L1 was upregulated in NSCLC cells and related to unfavorable prognosis in NSCLC patients. (**A and B)** Western blotting analysis compared PD-L1 levels between NSCLC cells and normal lung epithelial cells. **(C and D)** IHC analysis of PD-L1, PAS-CD31, used to detect the presence of VM in NSCLC tissue (black arrows: endothelium-dependent vessels, PAS-CD31+; red arrows: vasculogenic mimicry, PAS + CD31-; magnification, ×400). (**E and F)** Patient survival analysis demonstrated varying OS and DFS rates depended on PD-L1 expression levels

To investigate whether programmed death-ligand 1 (PD-L1) is overexpressed or underexpressed in NSCLC, Western blotting was employed to examine PD-L1 levels in all types of cells referred as before. The results suggested a remarkable overexpression of PD-L1 in A549, H1299, and PC9 cells. (Fig. 1A and B). Furthermore, a double-staining immunohistochemistry technique was employed to examine PD-L1 and PAS-CD31 expression in 100 NSCLC tissue samples. The results revealed that there were VM structures in cancerous tissues, with a higher prevalence in the group with PD-L1 expression (Fig. 1C and D). Notably, a significant association was observed between higher PD-L1 expression levels and shorter OS and DFS of the patients, as observed by immunohistochemistry analysis (Fig. 1E and F). Additionally, there was also a meaningful correlation between high or low PD-L1 expression and tumor size, TNM stage, lymph node metastasis, as well as poor prognosis in patients. (Table 3).

### VM formation was attenuated after Knockdown of PD-L1 in vitro

**Fig. 2:**
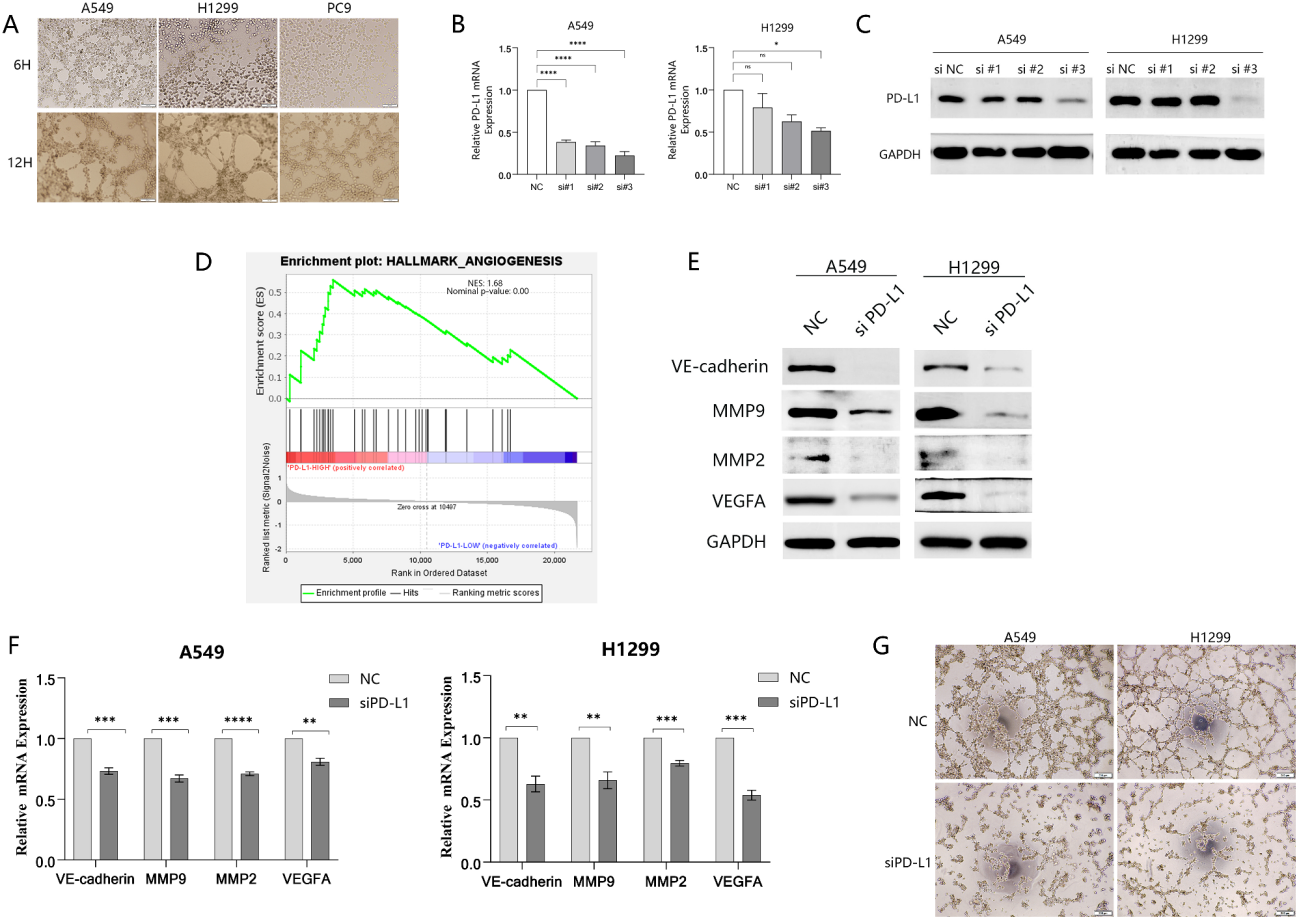
PD-L1 knockdown attenuated VM formation in vitro. **(A)** VM formation ability was assessed in all of three types cells. **(B and C )** The efficacy of PD-L1 knockdown using siRNA was evaluated through Western blotting and q-PCR. **(D)** The correlation between angiogenesis and PD-L1 levels was analyzed using the TCGA-Lung Adenocarcinoma (TCGA-LUAD) dataset, which comprised 465 samples. **(E and F)** VM-related genes’ expression in mRNA and protein levels was assessed through Western blotting and q-PCR analyses. **(G)** The number of VM structures decreased considerably following PD-L1 siRNA transfection

PD-L1 expression was examined in all three cell lines using Western blotting. Comparatively higher levels of PD-L1 were exhibited in A549 and H1299 cells (Fig. 1A). In addition, the tube-forming ability of these two cell lines was relatively obvious through tube-formation experiments (Fig. 2A). In summary, we selected these two cell lines to transfect siRNA-mediated PD-L1 knockdown, and verified the interference efficiency by Western blotting and q-PCR (Fig. 2B, C, and Supplementary Fig. 1A). Then we found that the #3 siRNA delivered the most effective knockdown efficiency. To explore the potential involvement of PD-L1 in NSCLC vascular, we conducted a bioinformatics analysis and observed significant activation of the angiogenic pathway in the overexpression group (Fig. 2D). Additionally, immunohistochemical analysis revealed PD-L1 positivity in 72 out of 100 NSCLC tissue cases, with 32 of these PD-L1 positive cases exhibiting vasculogenic mimicry (VM). A notable difference in VM occurrence was observed between the positive group with a 44% positive rate of VM and the negative group with 14%, highlighting that PD-L1 was closely related to VM formation (Fig. 1D). Knockdown of PD-L1 significantly reduced VM-related genes expression (MMP9, VE-cadherin, VEGFA, and MMP2) in A549 and H1299 cells (Fig. 2E, F, and Supplementary Fig. 1B). Subsequent results showed a significant reduction in VM structures of A549 and H1299 after knocking down PD-L1 (Fig. 2G and Supplementary Fig. 2A). Moreover, the presence of VM is remarkably correlated with multiple clinicopathological features (Table 4).

### Proliferation, invasion and metastasis of A549 and H1299 were inhibited following PD-L1 knockdown

**Fig. 3:**
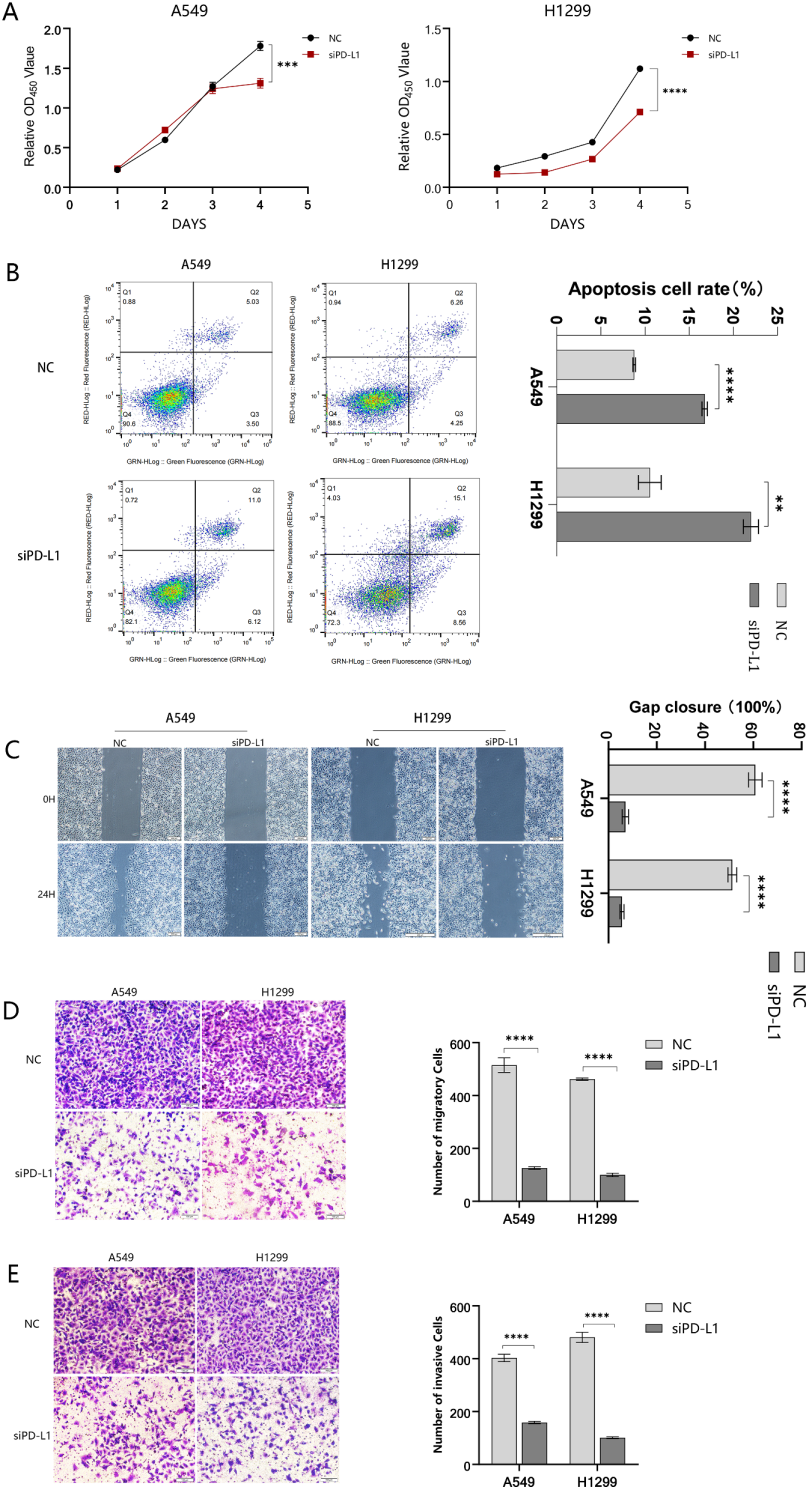
Proliferation, invasion and metastasis of A549 and H1299 were inhibited with PD-L1 knockdown. **(A)** CCK-8 assays assessed the proliferation capacity of A549 and H1299 following PD-L1 knockdown. **(B)** Flow cytometry was conducted to explore the rate of apoptosis in A549 and H1299 after transfection of siPD-L1. **(C, D and E)** Down-regulation of PD-L1 drastically inhibited migration and invasion ability in A549 and H1299 cells, as observed in all three assays (migration, invasion, and scratch healing)

As VM is thought to be linked to proliferation migration and invasion of malignant tumor cells, we explored the potential involvement of PD-L1 on these three functions in A549 and H1299. Our CCK8 experiments revealed the cell viability decreased considerably in the siPD-L1 group by the fourth day after PD-L1 knockdown (Fig. 3A). In addition, flow cytometry analysis demonstrated that suppression of PD-L1 increased the apoptosis rate of NSCLC cells, which coincided with the results of CCK8 experiments. (Fig. 3B). Finally, we used scratch and transwell assays to explore the effect of PD-L1 knockdown on the migratory ability of A549 and H1299. The results showed significant inhibition of their migration ability following PD-L1 knockdown (Fig. 3C, D and E).

### PD-L1 knockdown attenuated the expression of ZEB1 and EMT process

**Fig. 4:**
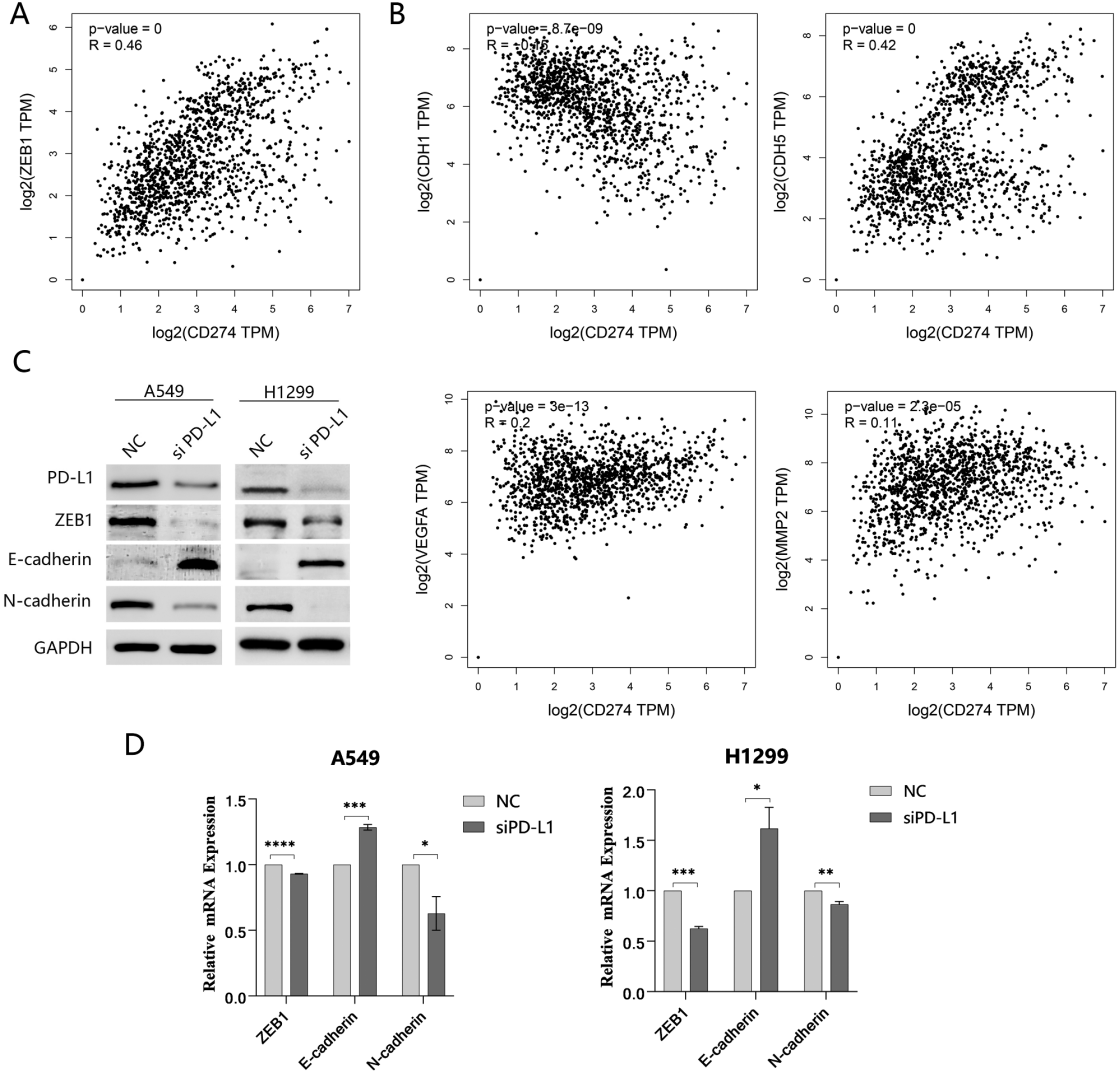
PD-L1 exhibited a relationship with ZEB1 and the EMT process. **(A and B)** Bioinformatic analysis revealed a correlation between PD-L1 and ZEB1 expression, as well as other EMT-related genes. **(C and D)** The expression of ZEB1 and EMT-related markers was assessed using Western blotting and q-PCR analysis in NSCLC cells transfected with PD-L1 siRNA

EMT is one of the numerous factors contributing to the formation of VM. While ZEB1 which is one of the transcripts that influence EMT occurrence exhibited an indispensable regulatory effect on this process. The results obtained by using a bioinformatics approach highlighted a meaningful correlation between PD-L1 and ZEB1 in expression levels, and the correlation coefficient was positive which indicated that a considerable positive correlation was found to exist between the expression levels of PD-L1 and the transcript ZEB1 (Fig. 4A). Meanwhile, PD-L1 was also associated with markers of EMT (Fig. 4B). Subsequently, we conducted investigations on EMT-related genes using Western blotting and q-PCR. Following PD-L1 knockdown, a notable reduction in ZEB1 and N-cadherin expression was observed, whereas E-cadherin exhibited the opposite trend (Fig. 4C, D, and Supplementary Fig. 1C).

**Cell lines construction and ZEB1 knockdown inhibited EMT occurrence, VM structures formation, migration and proliferation of NSCLC.**

**Fig. 5:**
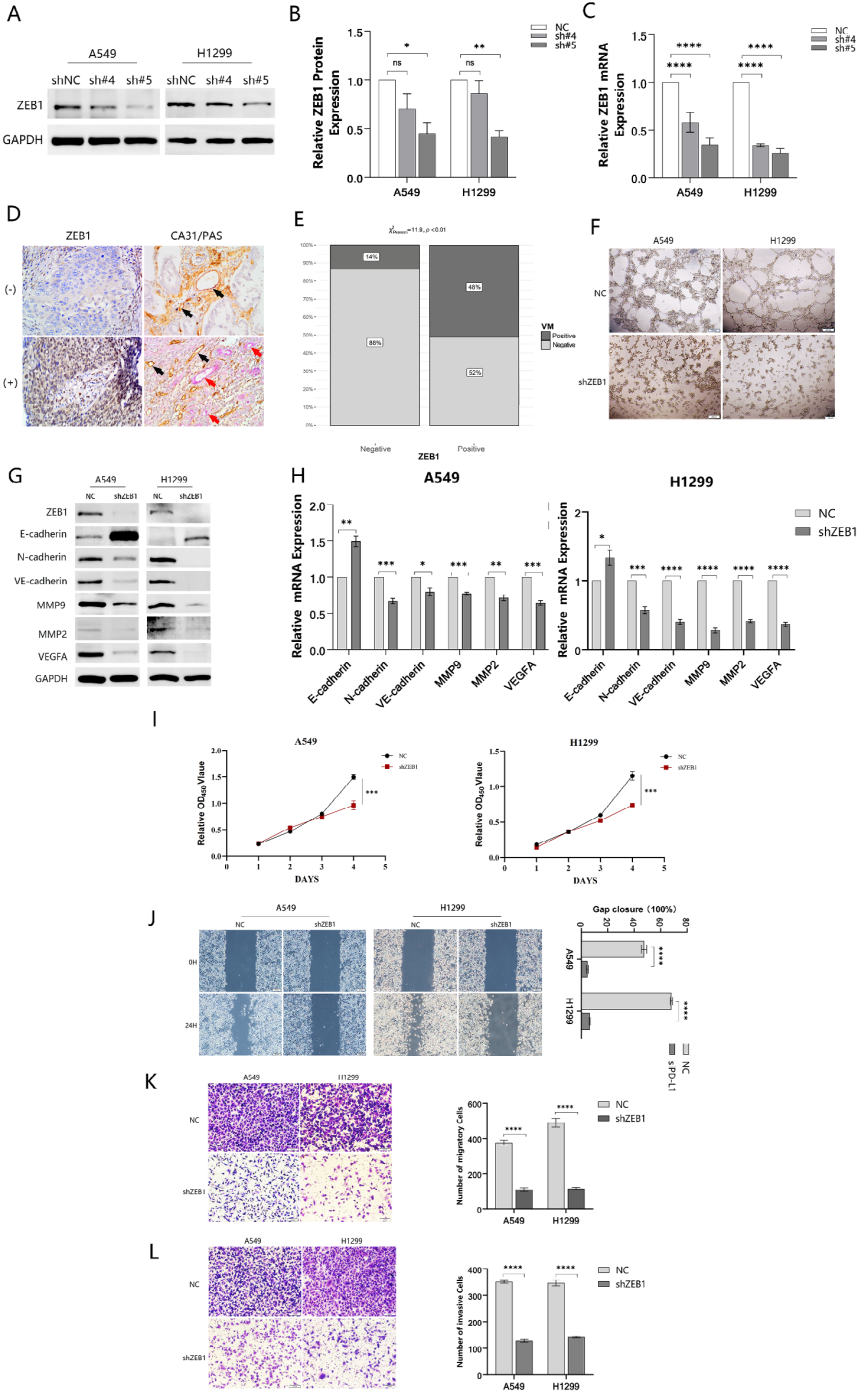
Cell lines construction and ZEB1 knockdown decreased EMT markers expression and VM structures formation in vitro. (**A, B and C)** Western blotting and q-PCR analysis of PD-L1 knockdown efficiency of shRNA. **(D and E)** Immunohistochemistry was employed to detect the presence of VM in both the ZEB1-positive and ZEB1-negative groups (magnification, ×400). **(F)** ZEB1 knockdown reduced the VM structure formation in vitro. **(G and H)** EMT-associated markers in NSCLC cells transfected with shRNA targeting ZEB1 were assayed at the protein and mRNA levels using Western blotting and q-PCR. **(I)** CCK8 assay demonstrated that the proliferation of NSCLCL cells was inhibited following ZEB1 knockdown. **(J, K and L)** Results from scratch healing assay, migration, and invasion experiments consistently demonstrated significant suppression of migratory and invasive capacities in A549 and H1299 cells upon ZEB1 knockdown

To elucidate the potential functions of ZEB1 in the EMT process and VM formation, we used lentivirus to establish a stable transducer strain of shZEB1. To determine the most efficient ZEB1 knockdown sequence for subsequent experiments, we employed Western blotting and q-PCR assays. The results demonstrated that the #5 shRNA exhibited the most significant efficiency (Fig. 5A, B, C). Additionally, Immunohistochemical analysis was conducted to investigate the correlation between ZEB1 and VM, reminiscent of the association observed between PD-L1 and VM. The results showed that among 100 cases of NSCLC specimens, ZEB1 positivity was observed in 64 cases, of which 31 cases exhibited VM occurrence. A significant difference in VM presence was observed in the ZEB1(+) group and the ZEB1(-) group. And the VM positivity rates in these two groups were 48% and 14%, respectively (Fig. 5D, E). The results of the tube formation experiment were the same as expected. That is, the suppression of ZEB1 resulted in a notable weakness in the tube formation ability, which is similar to the outcome observed upon knockdown of PD-L1 (Fig. 5F and Supplementary Fig. 2B). Subsequently, we performed assays to investigate the impact of ZEB1 knockdown on EMT-related genes, similar to our previous approach. And The results revealed that after ZEB1 knockdown, N-cadherin, VE-cadherin, MMP2, MMP9, VEGFA expression were significantly down-regulated, while E-cadherin expression showed the opposite trend (Fig. 5G, H, and Supplementary Fig. 1D). This result mirrored that observed after PD-L1 knockdown.

Furthermore, since VM is associated with proliferation and migration, we also explored whether ZEB1 has an effect on these two abilities of the A549 and 1299 cell lines. According to CCK8 results, it could be found that ZEB1 knockdown inhibited the proliferation capacity of both cell lines significantly (Fig. 5I). Wound-healing, migration, and invasion assays also yielded consistent results, demonstrating that there existed a meaningful inhibition on the migratory ability following ZEB1 knockdown (Fig. 5J, K and L).

**PD-L1 co-expressed with ZEB1 in NSCLC.**

**Fig. 6:**
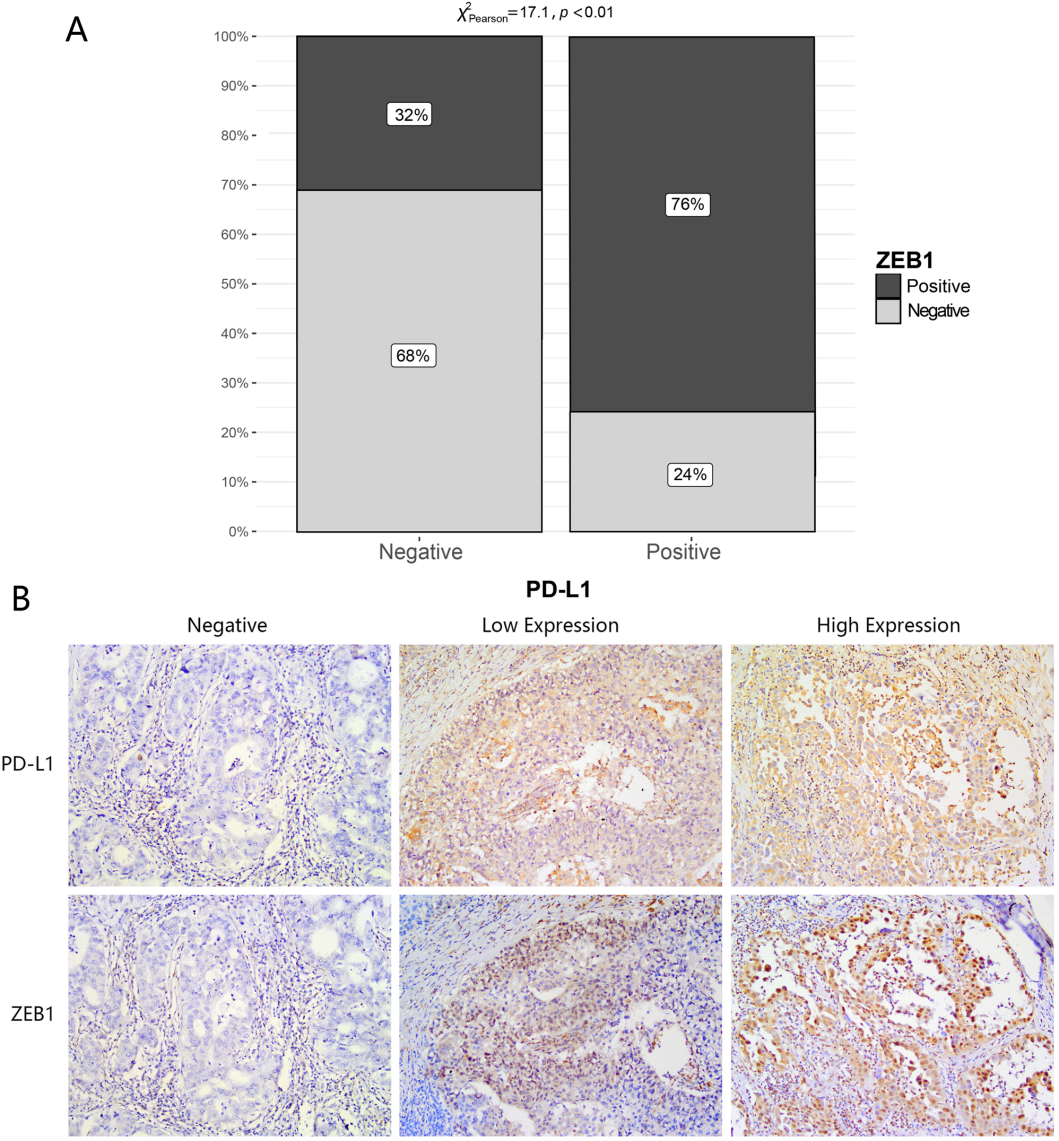
The distribution of PD-L1 exhibits concordance with the ZEB1 distribution. **(A)** Exploration of ZEB1 expression in distinct PD-L1 groups through immunohistochemistry. **(B)** Immunohistochemistry exhibited a remarkable concordance in the distribution patterns of PD-L1 and ZEB1 (magnification, ×200)

To further investigate the potential correlation existing between PD-L1 and ZEB1, we conducted immunohistochemistry assays. The results revealed a significant difference in the presence of ZEB1 between the PD-L1 positive (ZEB1-positive rate: 76%) and PD-L1 negative (ZEB1-negative rate: 32%) groups (Fig. 6A). The distribution of PD-L1 and ZEB1 was examined through immunohistochemical staining of consecutive sections from the wax block samples. Additionally, our results demonstrated a strong correlation between the distributions of PD-L1 and ZEB1 (Fig. 6B).

## Discussion

Lung cancer ranks second as the most prevalent malignancy globally. Meanwhile, it has the first mortality rate and the second incidence. Non-small cell lung cancer (NSCLC) comprises approximately 85% of lung cancer cases. Notably, recurrence and metastasis are considered to be the primary contributors to mortality among patients with lung cancer. In addition, once patients become resistant to conventional chemotherapy and radiotherapy, then anti-angiogenic therapy will become an important treatment for this group of patients. However, in recent years, the cure rate of antiangiogenic therapy is not significant. Additionally, there is a hypothesis saying the anti-angiogenic treatment may promote the structures of vasculogenic mimicry (VM) formation, then resulting in further metastasis of tumors. The essential role of VM in regulating cancer initiation and progression has been well-documented in academic studies. This finding has further enhanced our understanding of the significance of VM in tumor angiogenesis and metastasis. However, how vasculogenic mimicry arises is still unknown. Hence, there is an urgency to explore the mechanism of VM generation for the purpose of providing cancer treatment with new strategies.

PD-L1, an immunomodulatory ligand in the B7/CD28 family, has been shown to be closely associated with EMT genes such as E-cadherin, N-cadherin, VE-cadherin, and others. Some studies have also shown that angiogenesis in tumor tissues is significantly promoted when PD-L1 is overexpressed.

This study elucidates the significant influence of PD-L1 on VM formation in NSCLC through multiple levels of investigation. Firstly, we caught a remarkable link between PD-L1 and VM, as well as epithelial-mesenchymal transition (EMT), which is involved in VM formation. Additionally, the bioinformatic analysis uncovered a positive correlation of PD-L1 and ZEB1. Secondly, we observed a significant upregulation of PD-L1 in NSCLC cells (A549, H1299 as well as PC9) compared to normal lung epithelial cells (BEAS-2B). The immunohistochemistry results revealed a pronounced decrease in OS and DFS among the patients who exhibited heightened PD-L1 levels. Conversely, patients with low PD-L1 expression demonstrated improved OS and DFS. In addition, for the purpose of investigating the role of PD-L1 in NSCLC, we conducted multiple studies utilizing NSCLC cell lines with knockdown of PD-L1 in vitro. Our study revealed that PD-L1 exhibited a positive correlation with ZEB1, VM, and related markers of EMT. Specifically, we found that PD-L1 was positively correlated with ZEB1, N-cadherin, MMP9, VE-cadherin, and VEGFA, while it showed a negative correlation with E-cadherin. Furthermore, our research demonstrated that reducing PD-L1 levels effectively suppressed VM structure formation in NSCLC and reduced the biological activities of cancer cells. We discovered that vasculogenic mimicry formation and EMT processes were inhibited by the knockdown of ZEB1 by lentivirus. Additionally, immunohistochemical analysis indicated a co-expression of PD-L1 and ZEB1. These findings provide preliminary evidence demonstrating that VM formation may be promoted in NSCLC through the EMT process which is triggered by ZEB1.

## Conclusions

In conclusion, our study indicates that targeting PD-L1 has the potential to be an innovative therapeutic approach for inhibiting VM formation and enhancing the effectiveness of antiangiogenic therapy. We found that knocking down PD-L1 can effectively block the EMT process by inhibiting ZEB1, thereby leading to the suppression of VM formation. This suggests that by targeting PD-L1, we may be able to overcome resistance to current treatments and improve treatment outcomes.

### Electronic supplementary material

Below is the link to the electronic supplementary material.

Supplementary Material 1

Supplementary Material 2

Supplementary Material 3

## Data Availability

All data generated or analyzed during this study have been included in this manuscript.

## Abbreviations

NSCLC: non-small cell lung cancer
PD-L1: programmed death protein ligand-1
EMT: epithelial-mesenchymal transition
VM: vasculogenic mimicry
NC: normal control
Si-PD-L1: siRNA-PD-L1
Sh-ZEB1: shRNA-ZEB1
WB: Western blotting
q-PCR: quantitative real-time polymerase chain reaction
CCK8: cell counting kit-8 assay
