# Supplementary figures and images for: PD-L1 knockdown suppresses vasculogenic mimicry of non-small cell lung cancer by modulating ZEB1-triggered EMT
Source: BMC Cancer. 2024 May 23;24:633. doi: 10.1186/s12885-024-12390-8 (PMC11118770; doi:10.1186/s12885-024-12390-8)

A

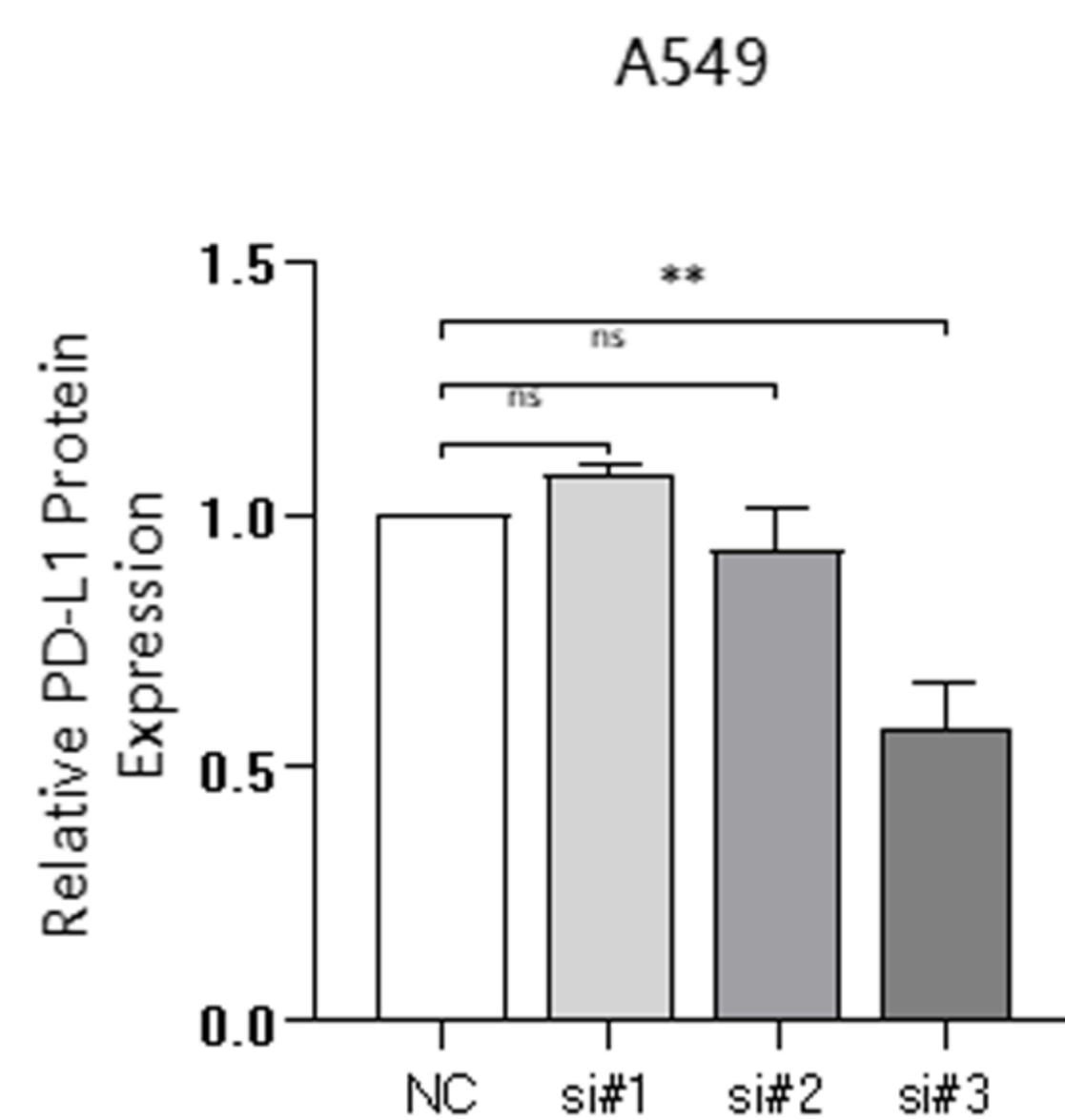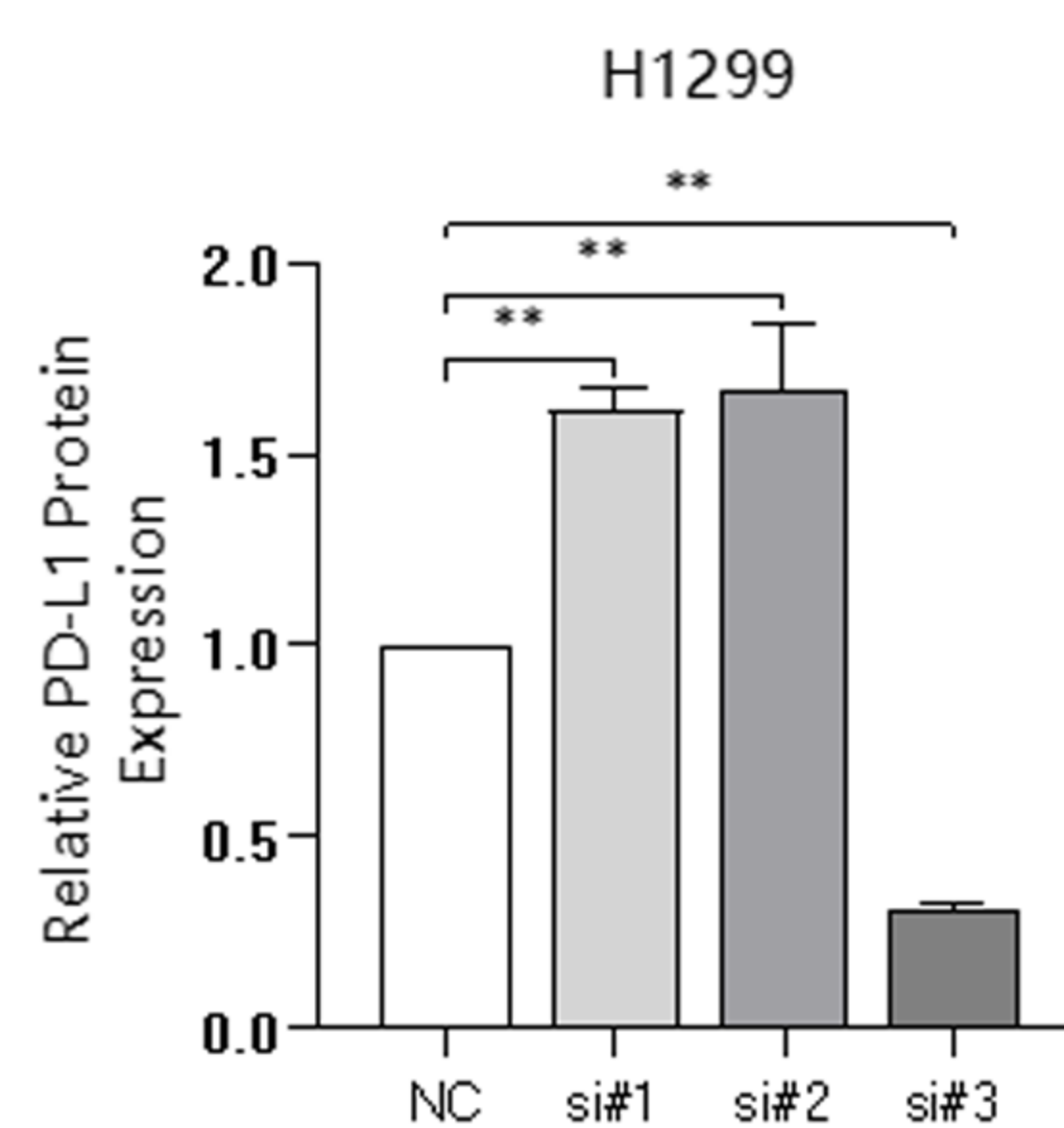

B

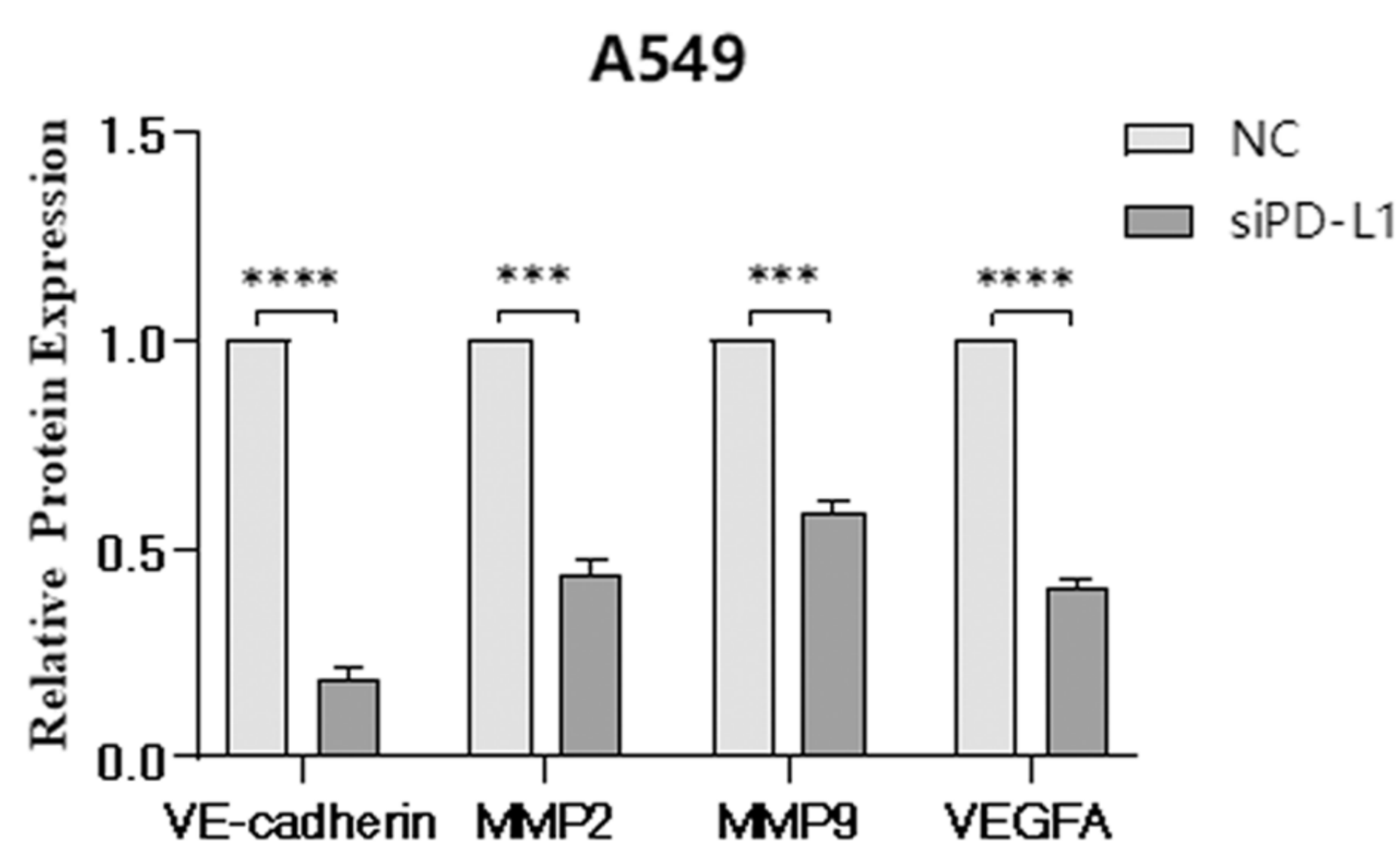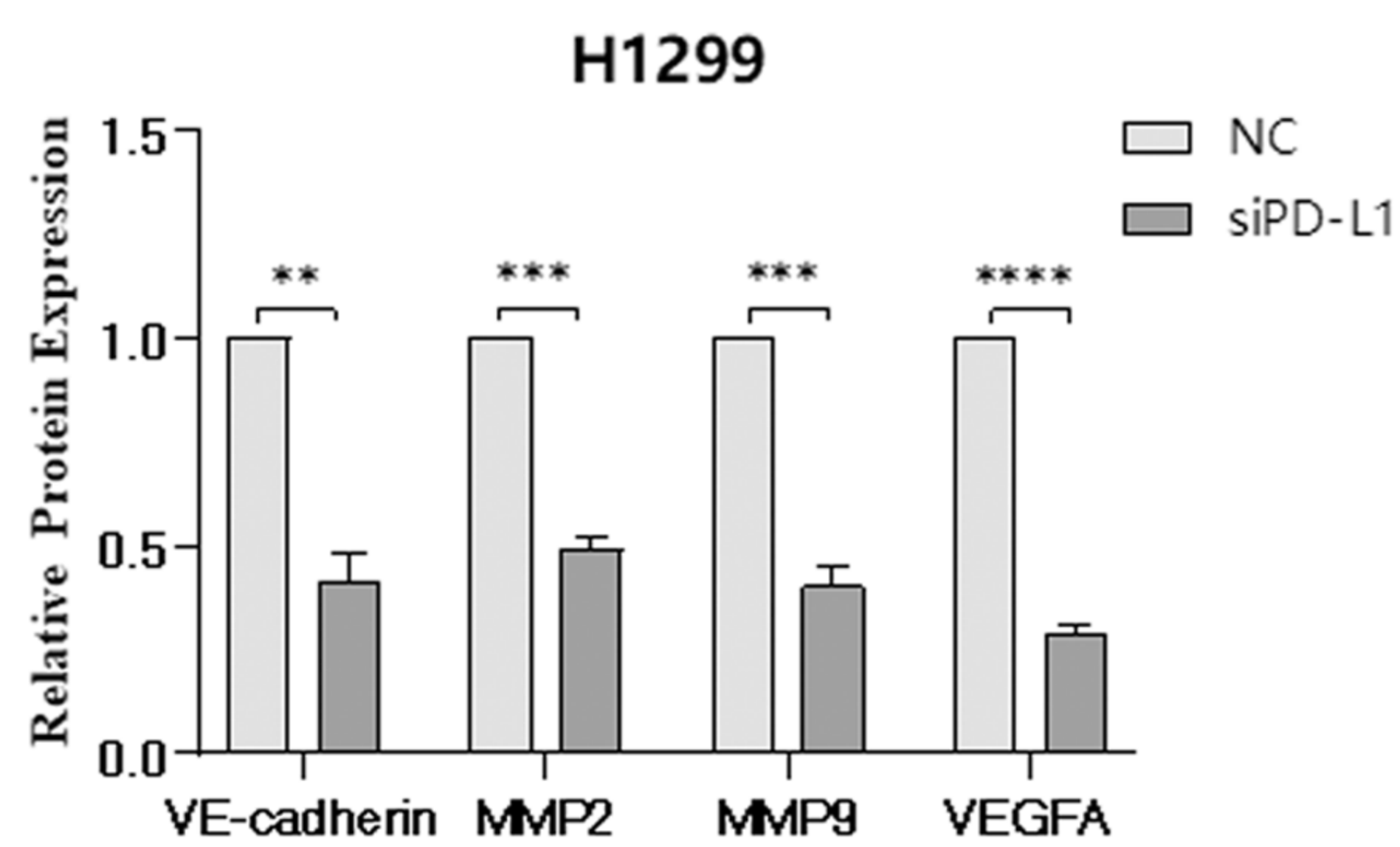

C

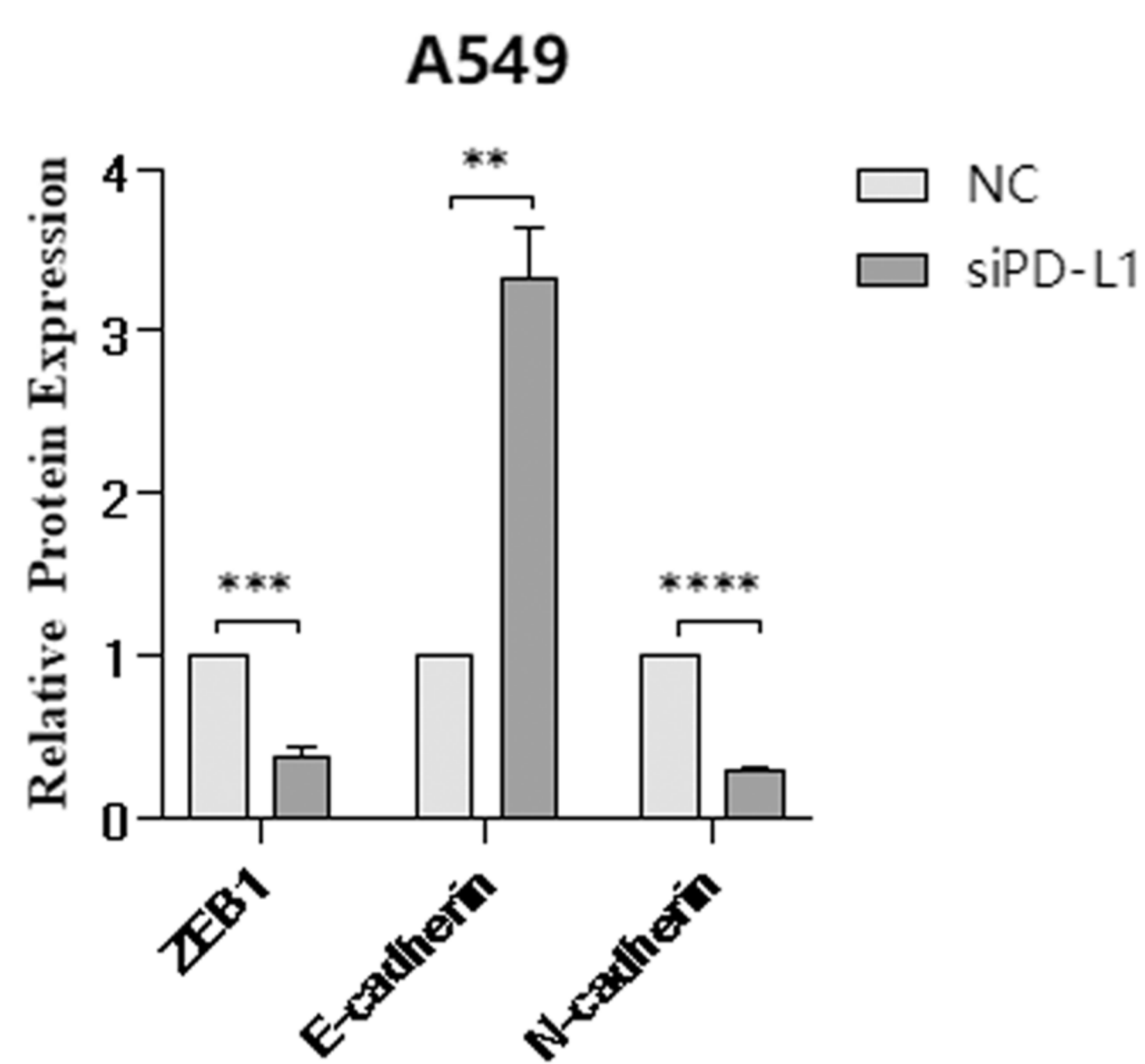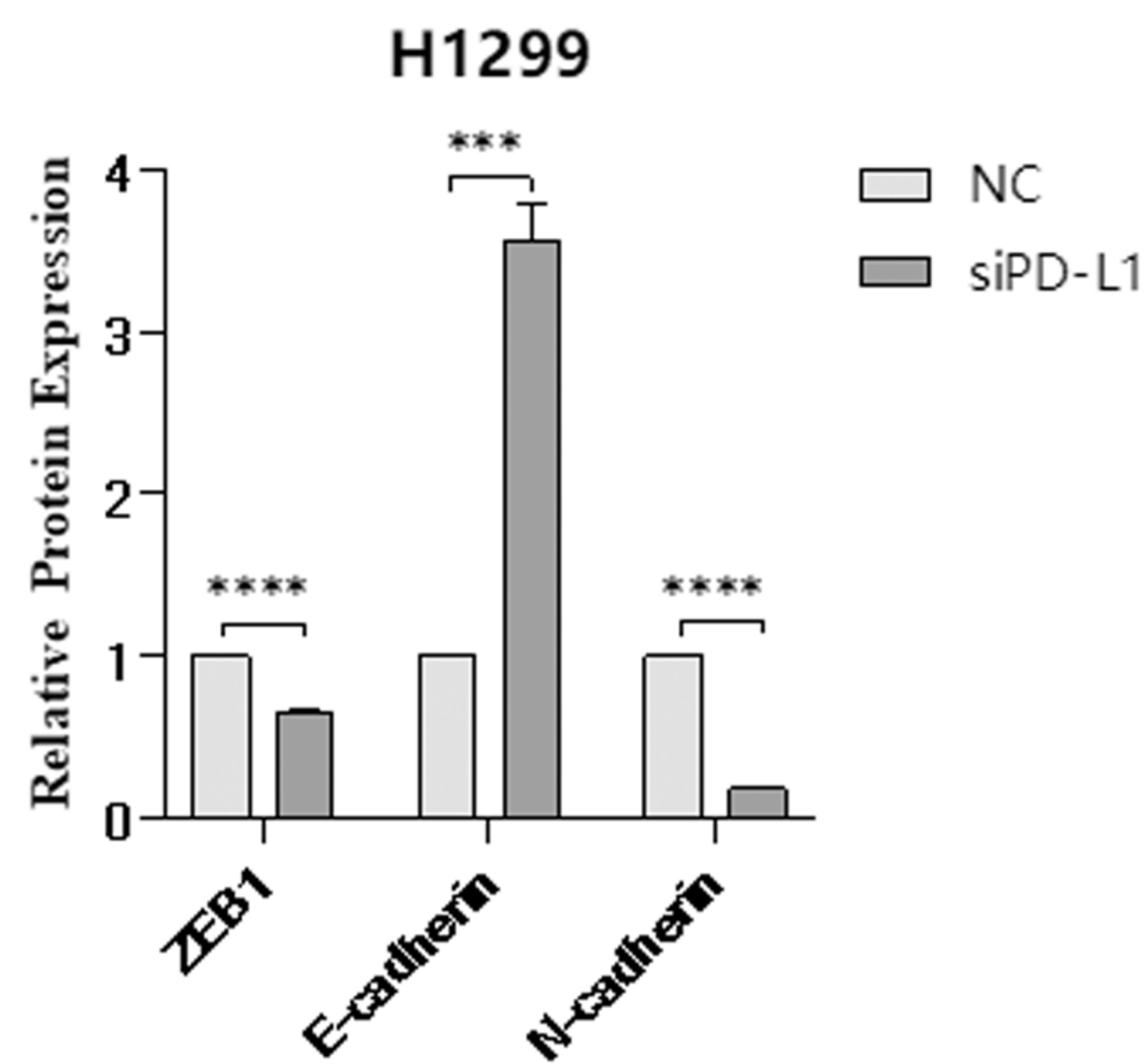

D

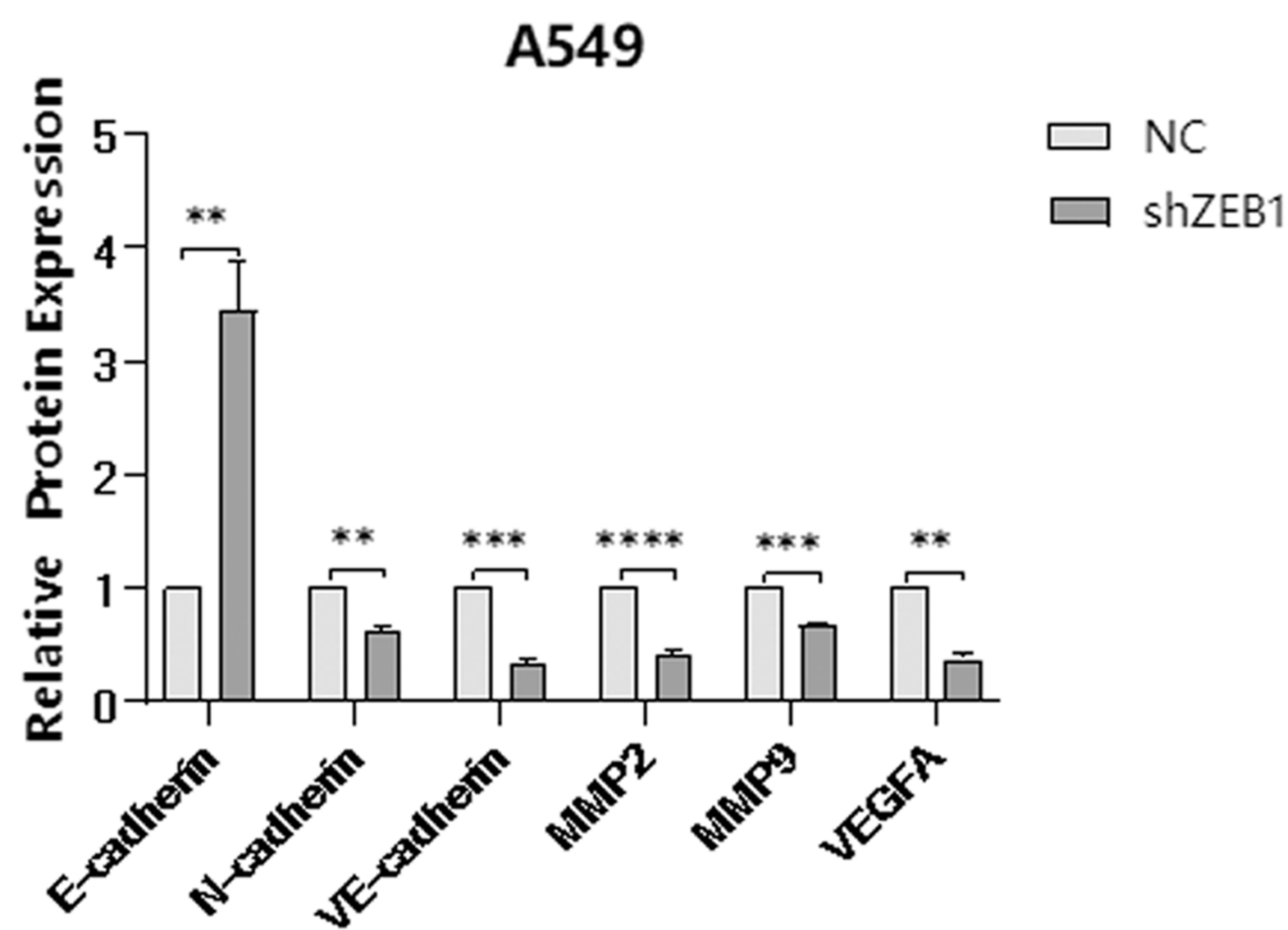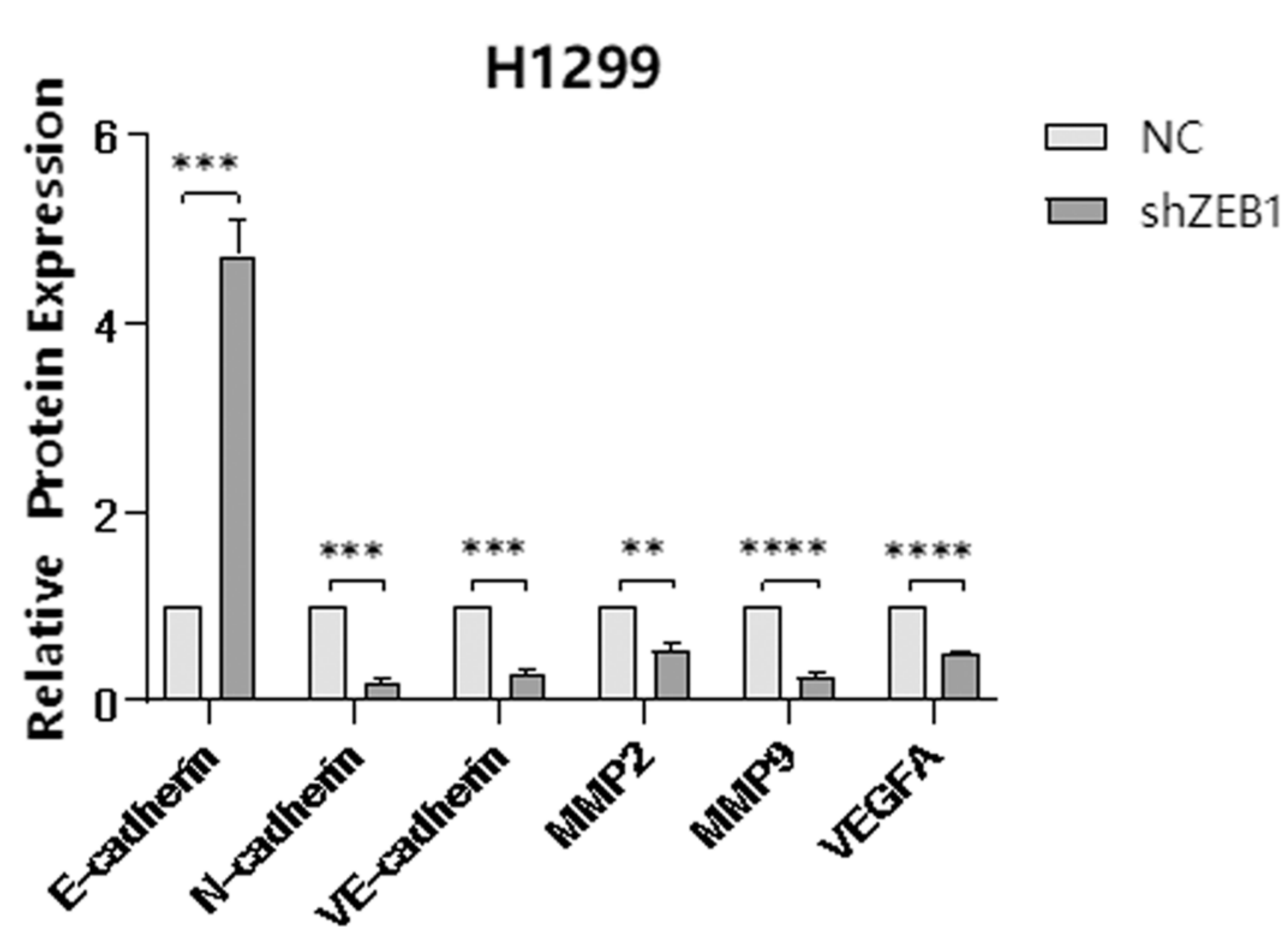

Supplement: Supplementary file 1 — Supplementary Material 1 [file 12885_2024_12390_MOESM1_ESM.pdf]

**FIG. A**

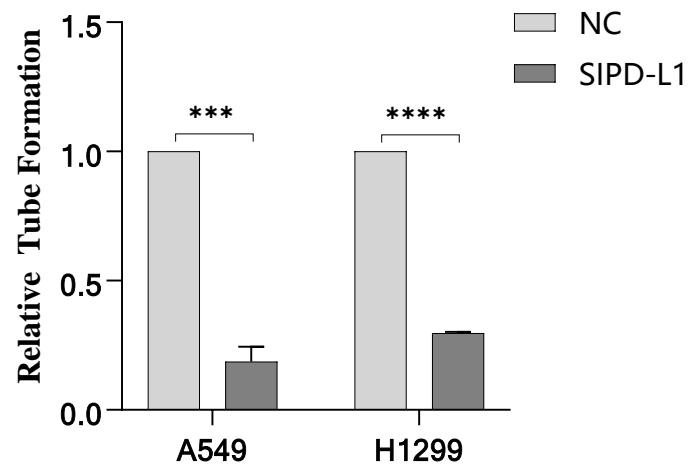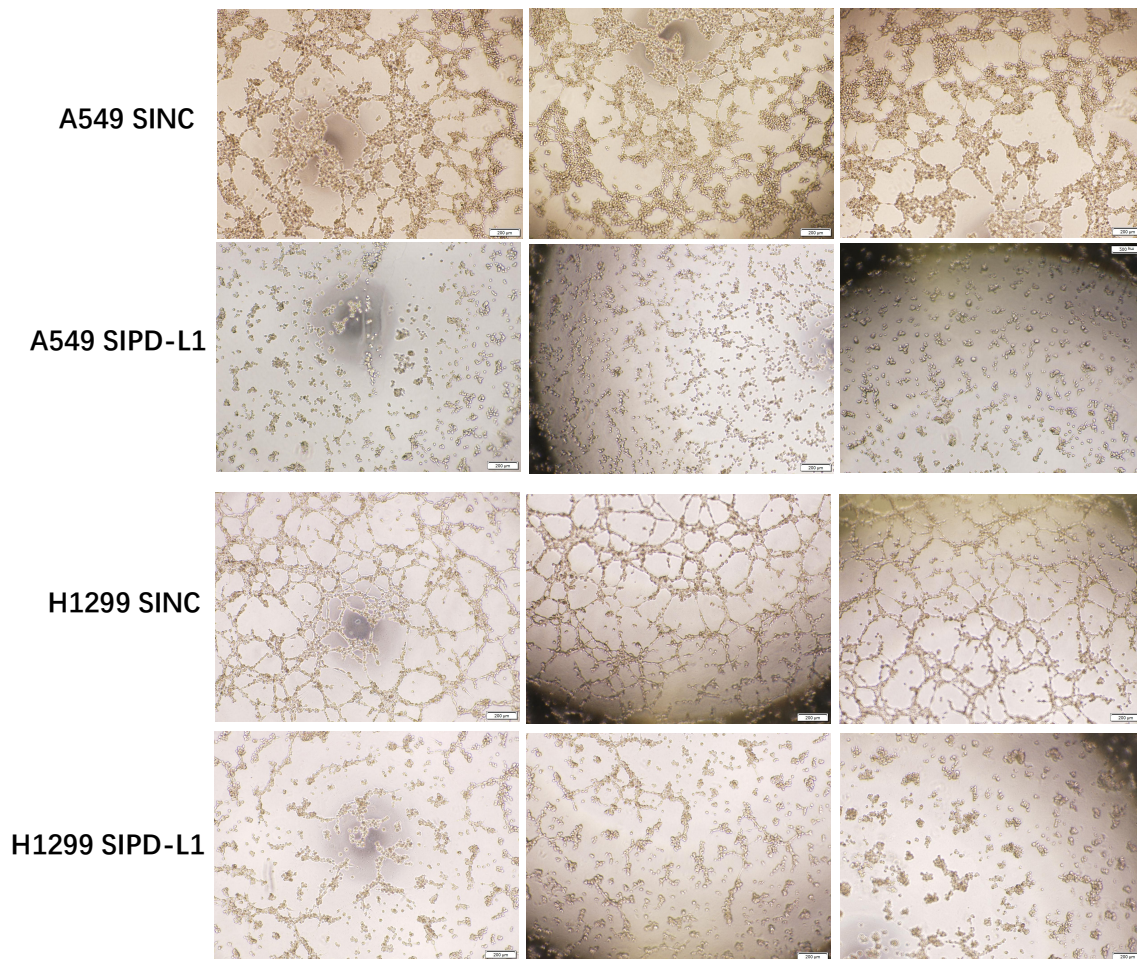

**FIG. B**

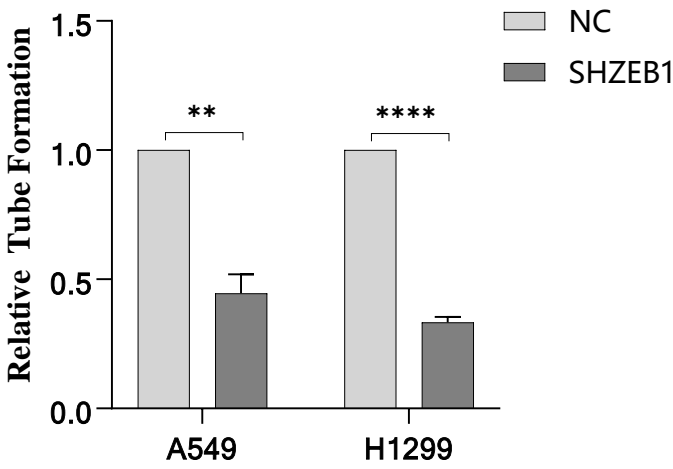

A549 SHNC

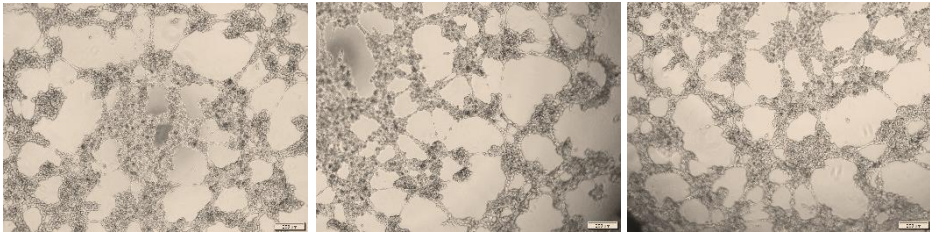

A549 SHZEB1

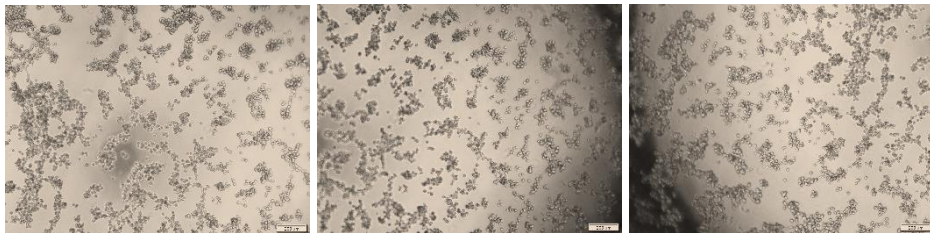

H1299 SHNC

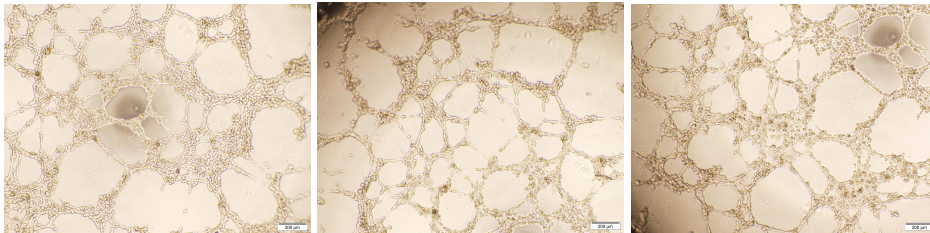

H1299 SHZEB1

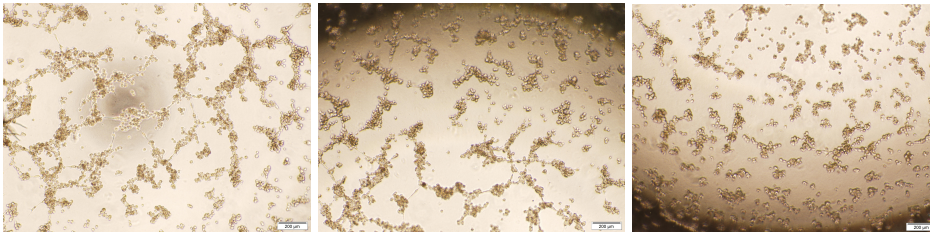

Supplement: Supplementary file 3 — Supplementary Material 3 [file 12885_2024_12390_MOESM3_ESM.pdf]
