# Supplementary material for: PD-L1 knockdown suppresses vasculogenic mimicry of non-small cell lung cancer by modulating ZEB1-triggered EMT
Source: BMC Cancer. 2024 May 23;24:633. doi: 10.1186/s12885-024-12390-8 (PMC11118770; doi:10.1186/s12885-024-12390-8)
Supplement: Supplementary file 2 — Supplementary Material 2 [file 12885_2024_12390_MOESM2_ESM.pdf]

Figure1 A

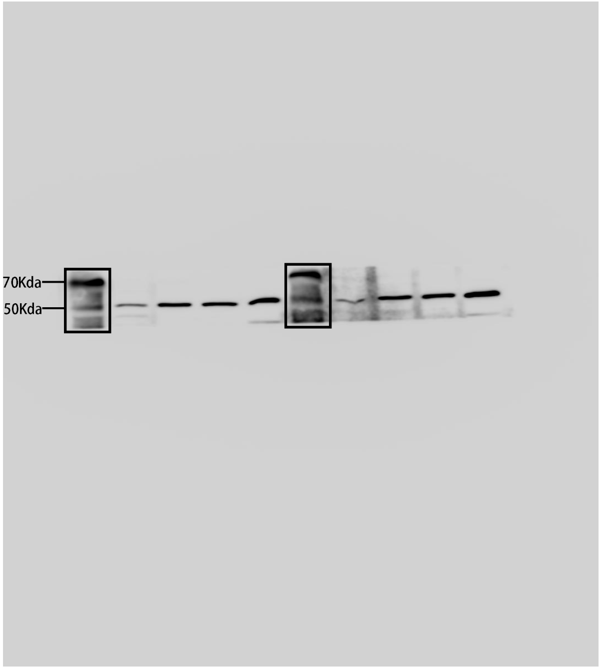

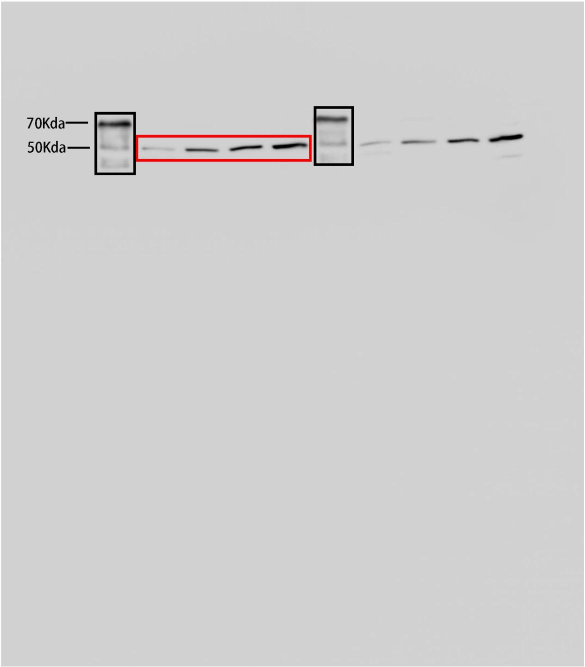

Original western blot image of PD-L1.

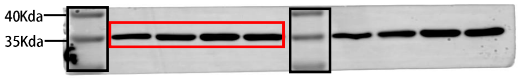

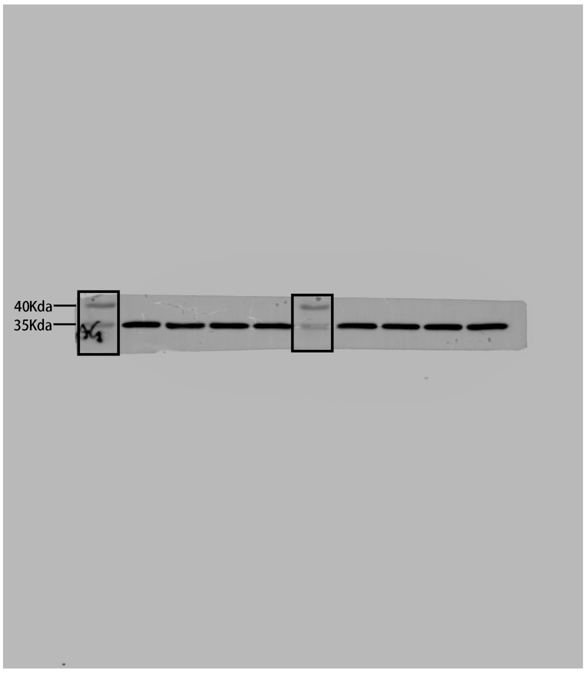

Original western blot image of GAPDH.

Figure2 C

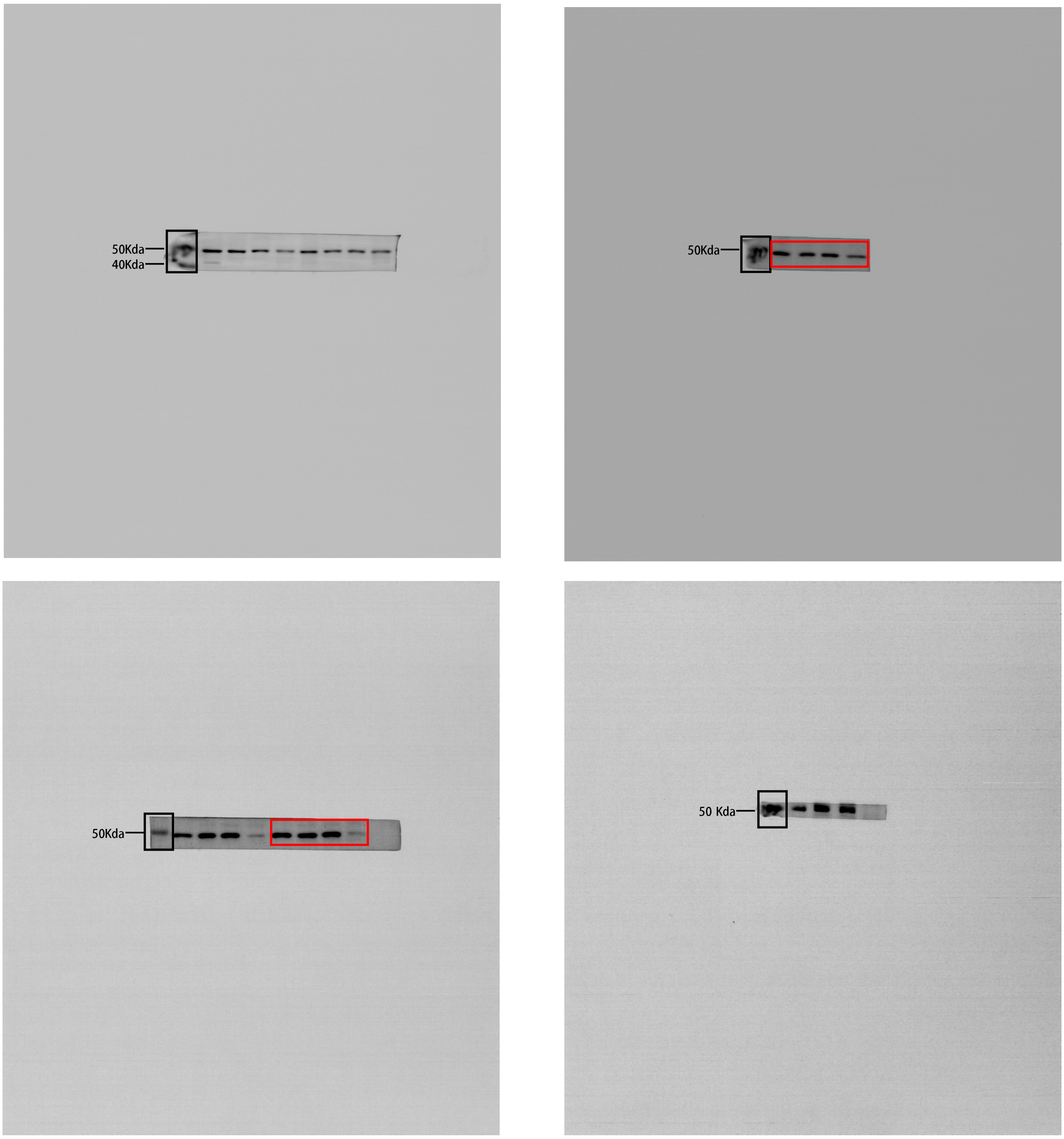

Original western blot image of PD-L1 siRNA, this band was cut in half for the other proteins before hybridization with the antibody.

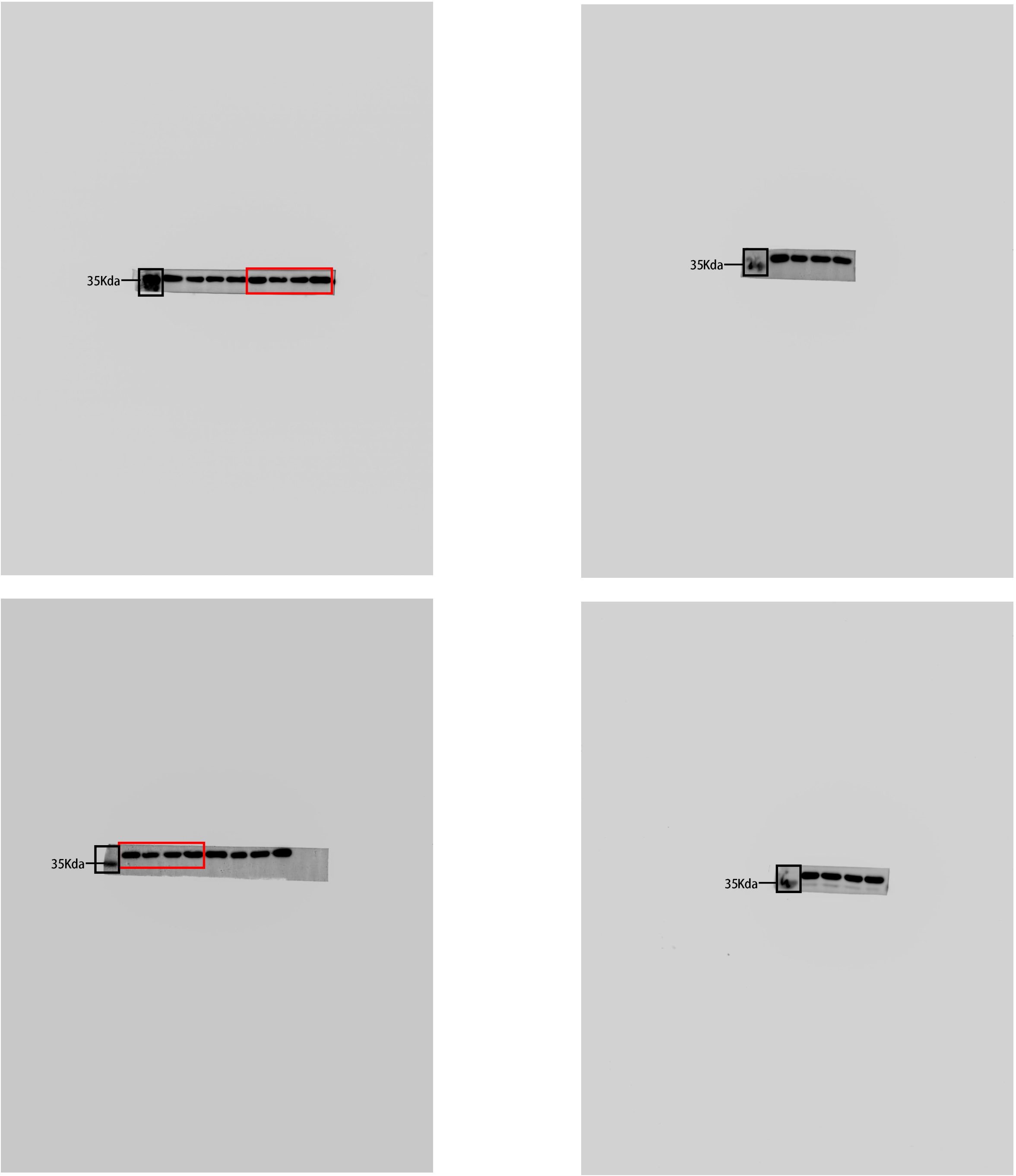

Original western blot image of GAPDH (PD-L1-siRNA), this band was cut in half for the other proteins before hybridization with the antibody.

Figure2 G

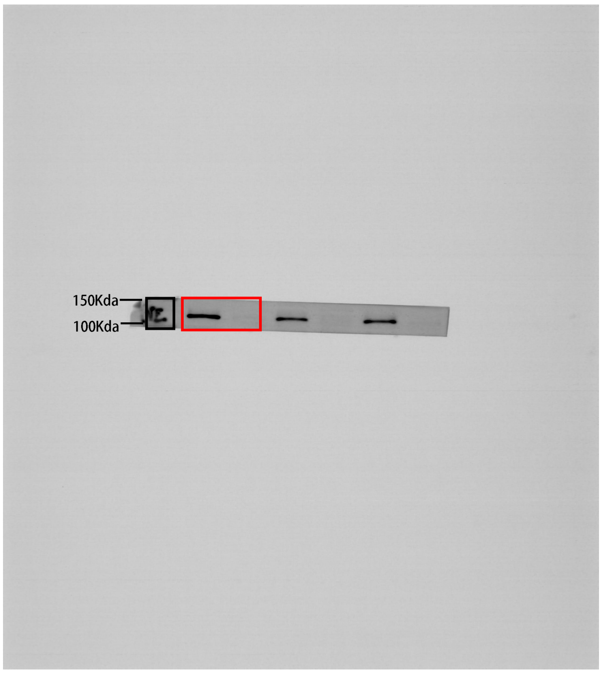

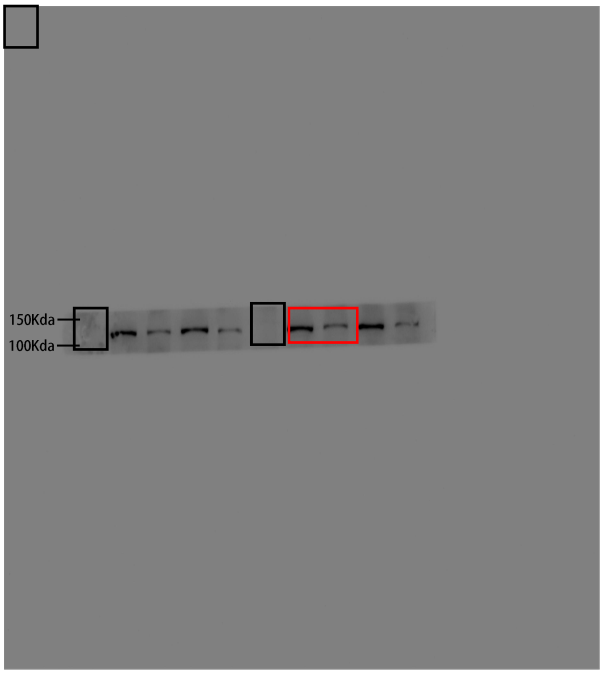

Original western blot image of VE-cadherin in A549 (left) and H1299 (right), this band was cut for the other proteins before hybridization with the antibody.

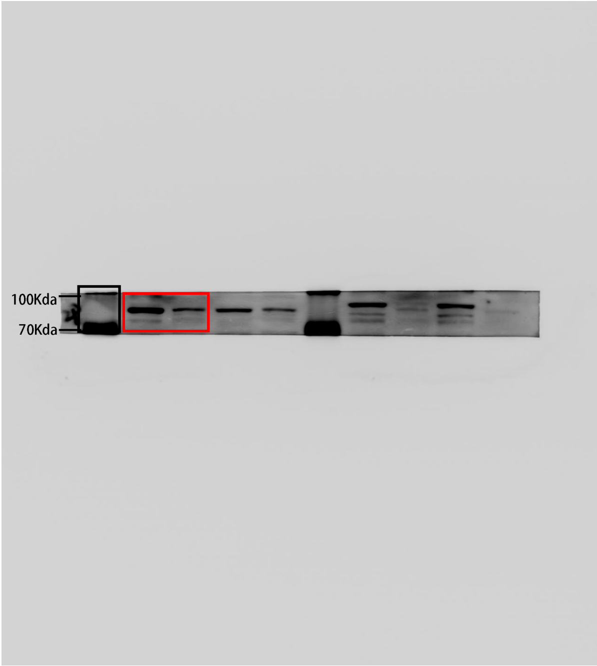

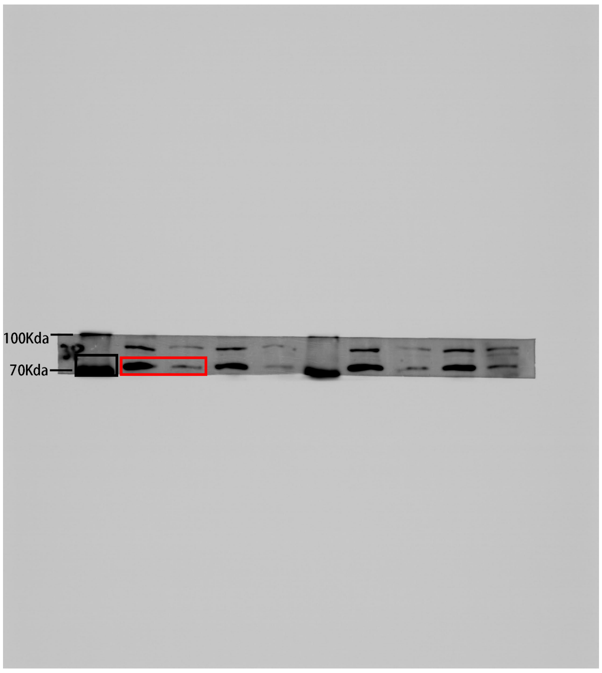

Original western blot image of MMP9 in A549 (left) and H1299 (right).

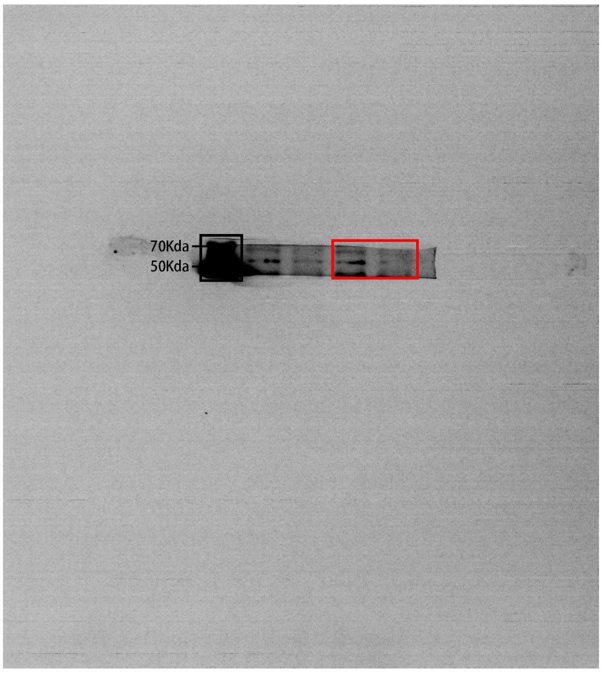

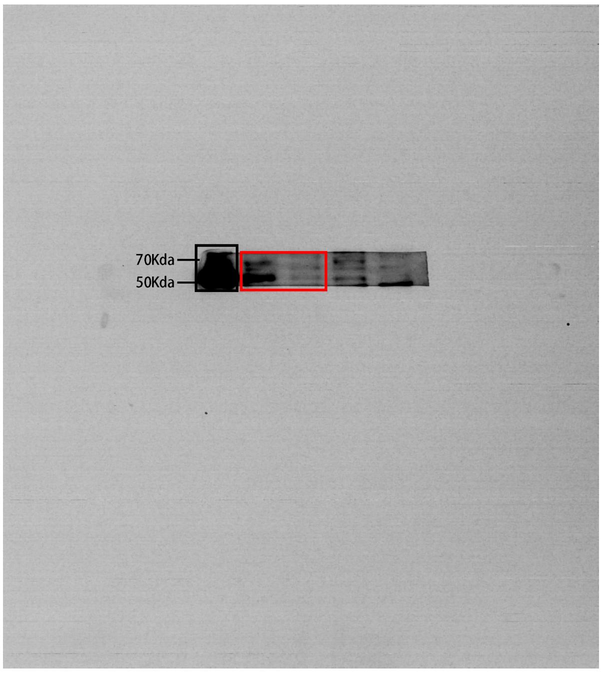

Original western blot image of MMP2 in A549 (left) and H1299 (right), this band was cut in half for the other proteins before hybridization with the antibody.

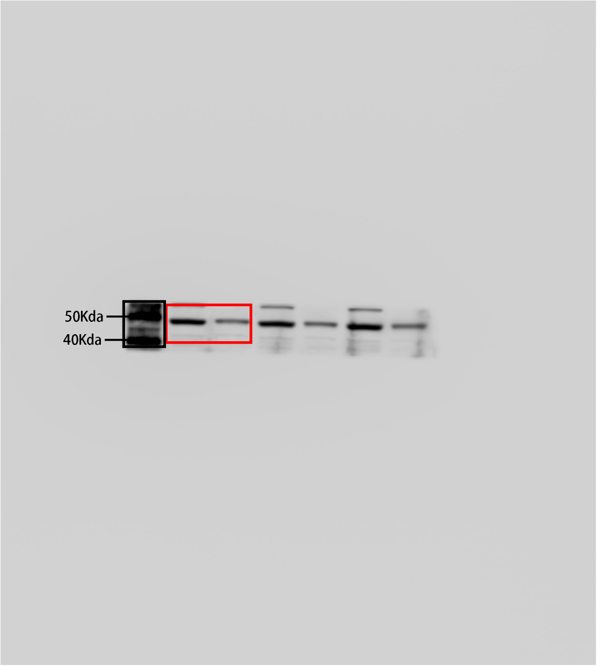

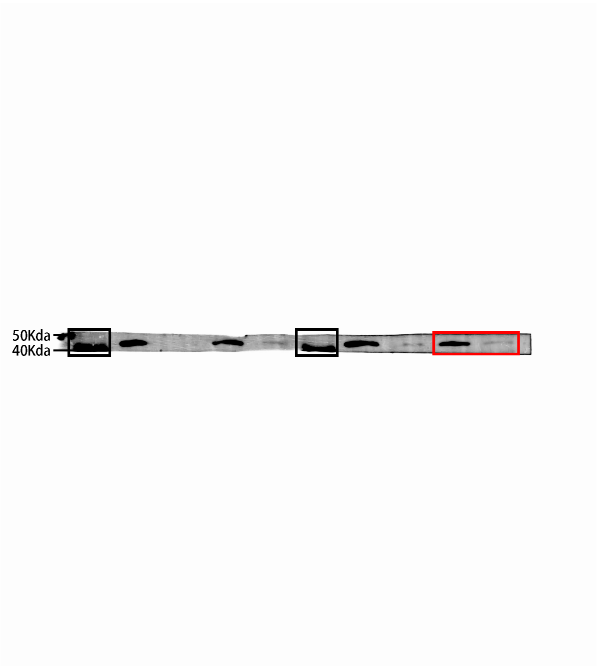

Original western blot image of VEGFA in A549 (left) and H1299 (right), this band was cut for the other proteins before hybridization with the antibody.

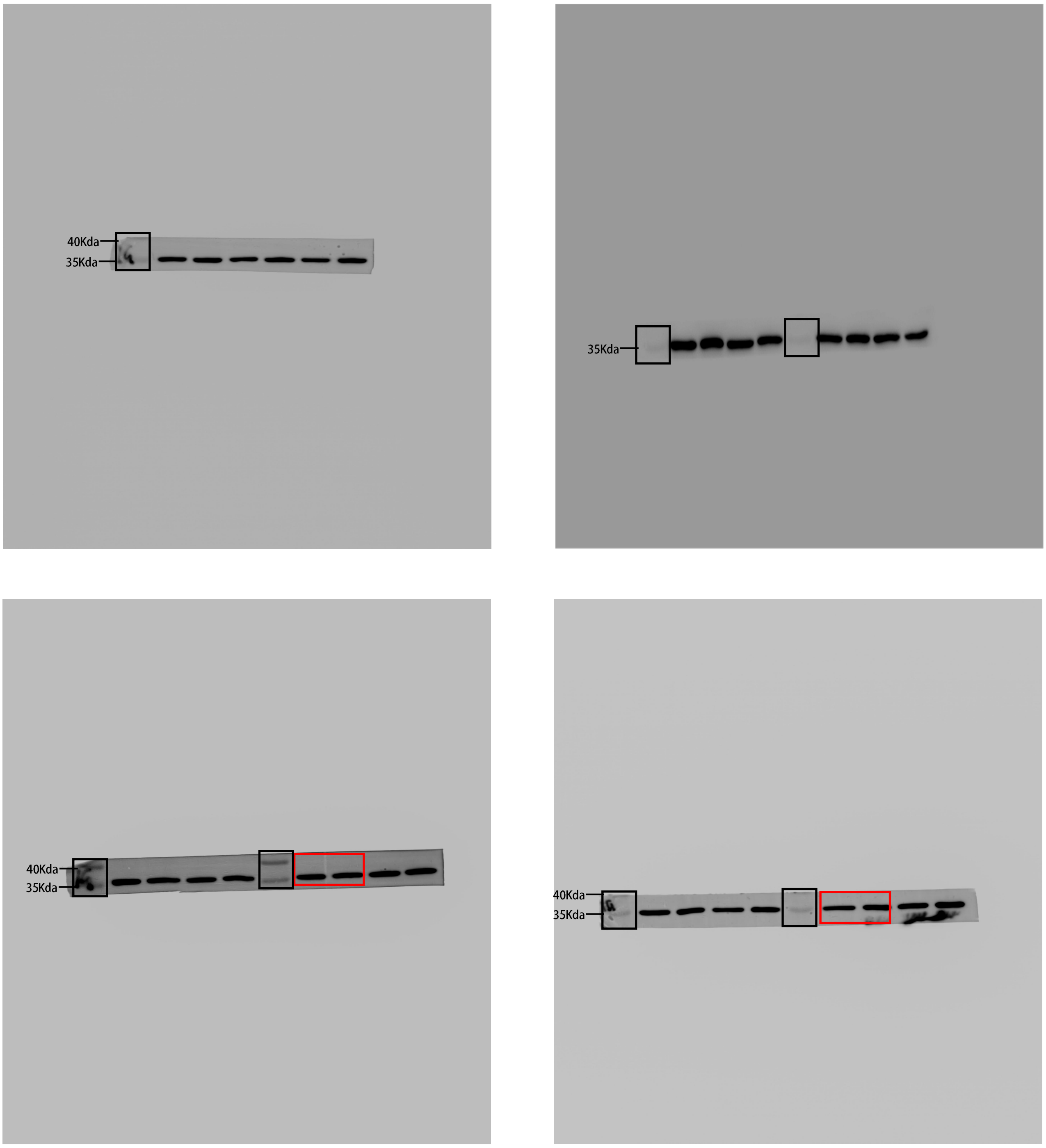

Original western blot image of GAPDH in A549 (left) and H1299 (right), this band was cut for the other proteins before hybridization with the antibody.

Figure4 C

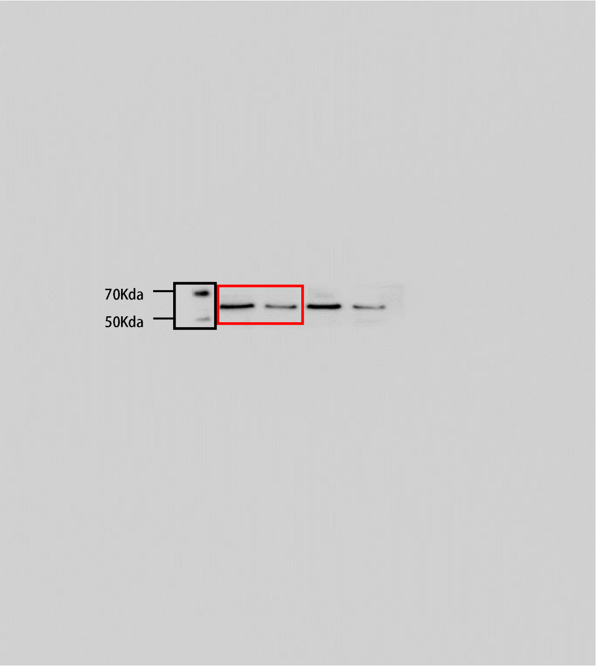

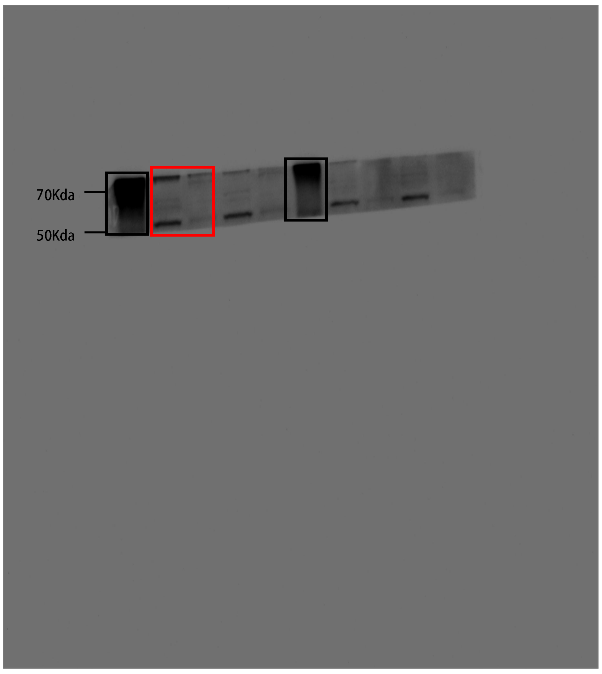

Original western blot image of PD-L1 in A549 (left) and H1299 (right), this band was cut in half for the other proteins before hybridization with the antibody.

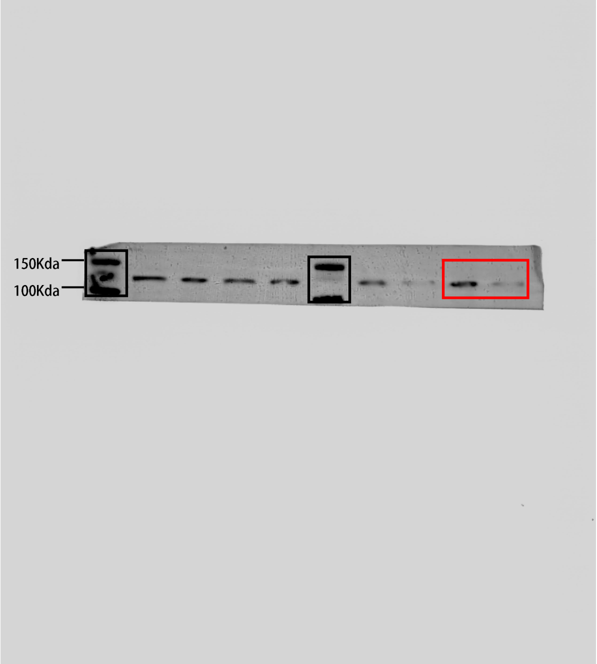

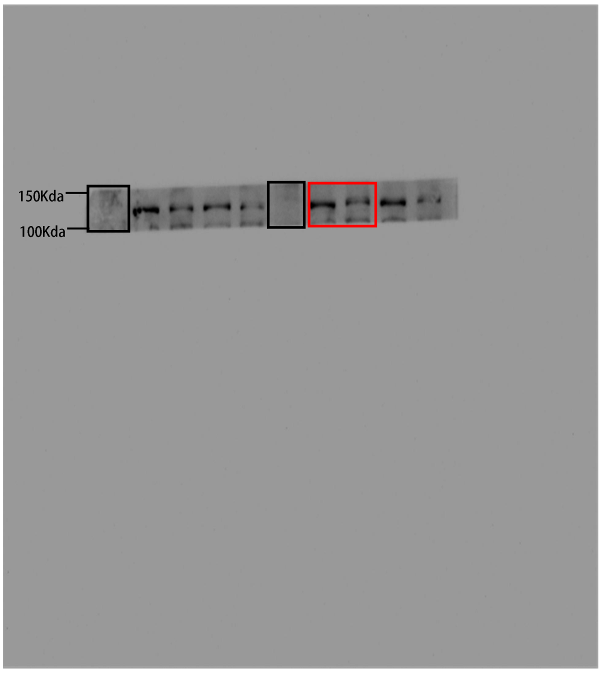

Original western blot image of ZEB1in A549 (left) and H1299 (right).

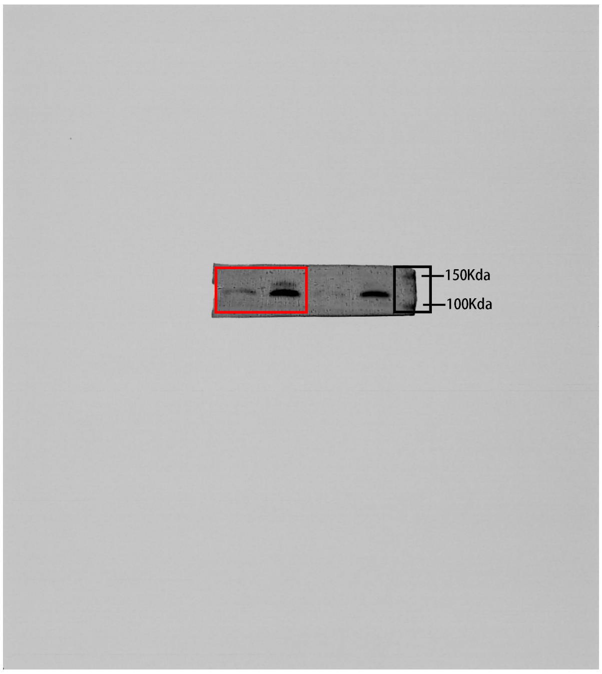

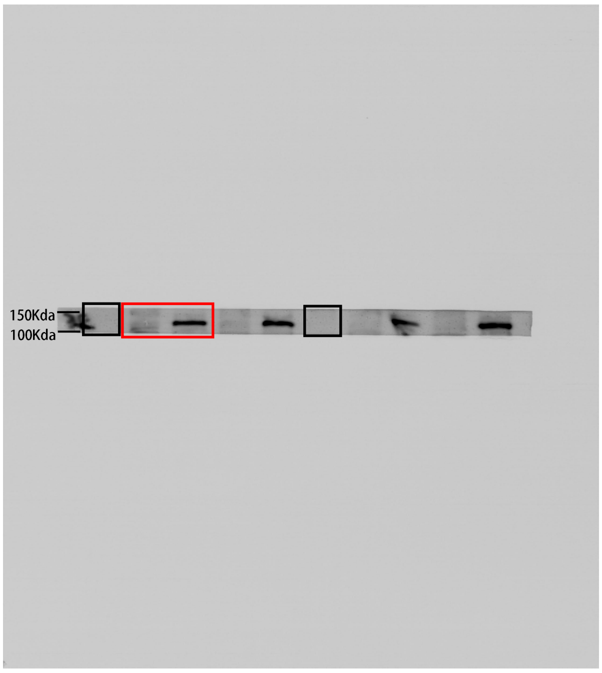

Original western blot image of E-cadherin A549 (left) and H1299 (right), this band was cut in half for the other proteins before hybridization with the antibody.

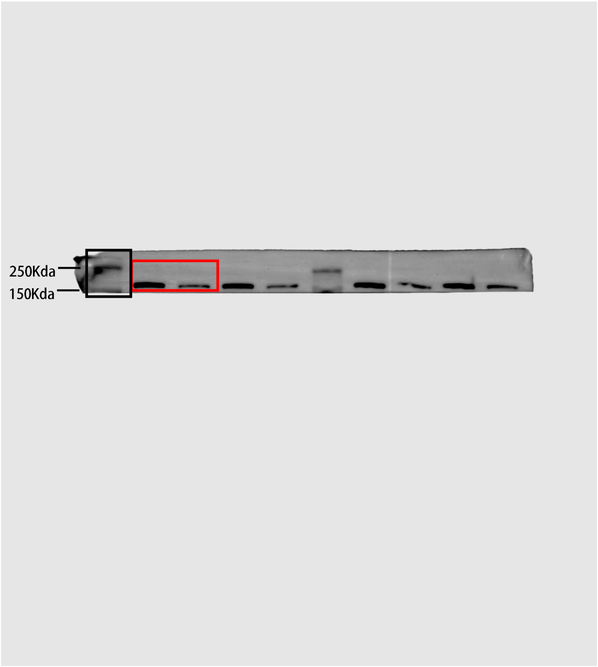

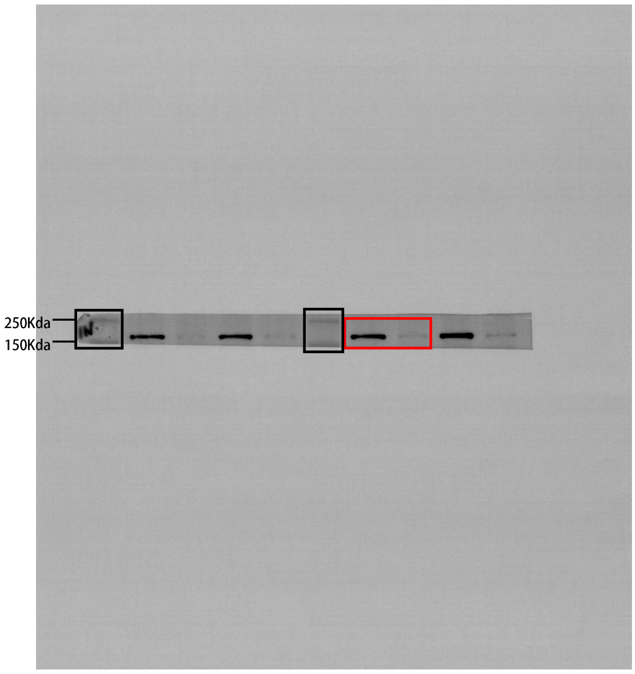

Original western blot image of N-cadherin A549 (left) and H1299 (right).

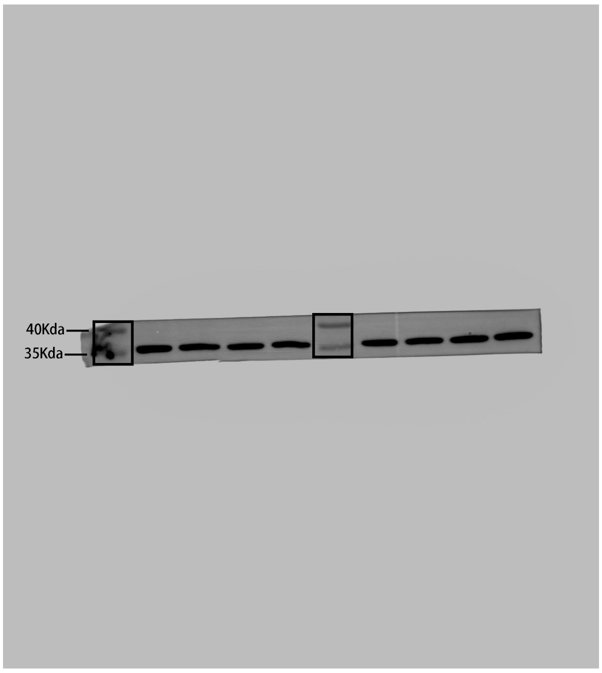

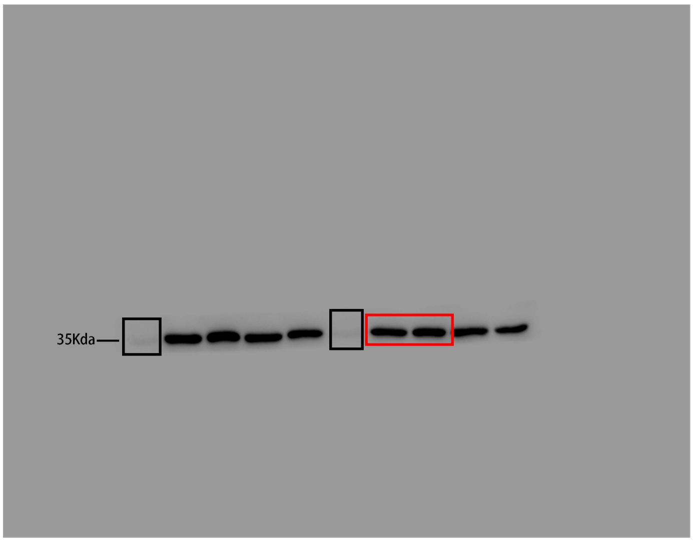

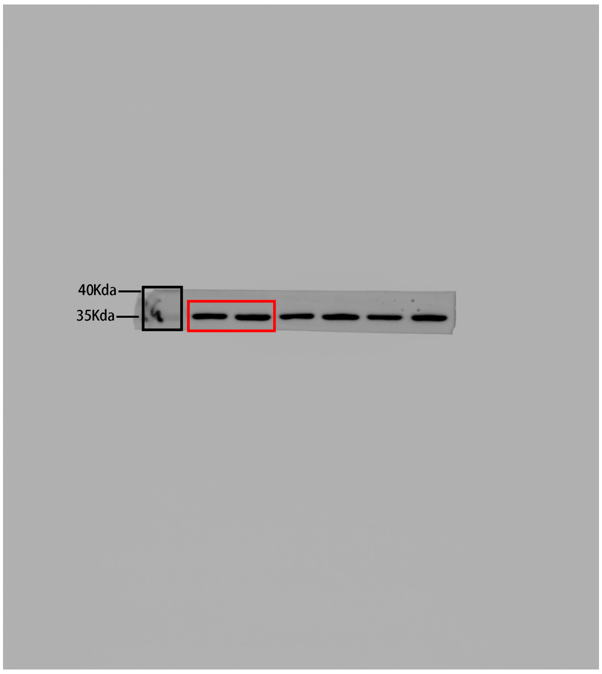

Original western blot image of GAPDH A549 (left) and H1299 (right), this band was cut for the other proteins before hybridization with the antibody.

Figure5 A

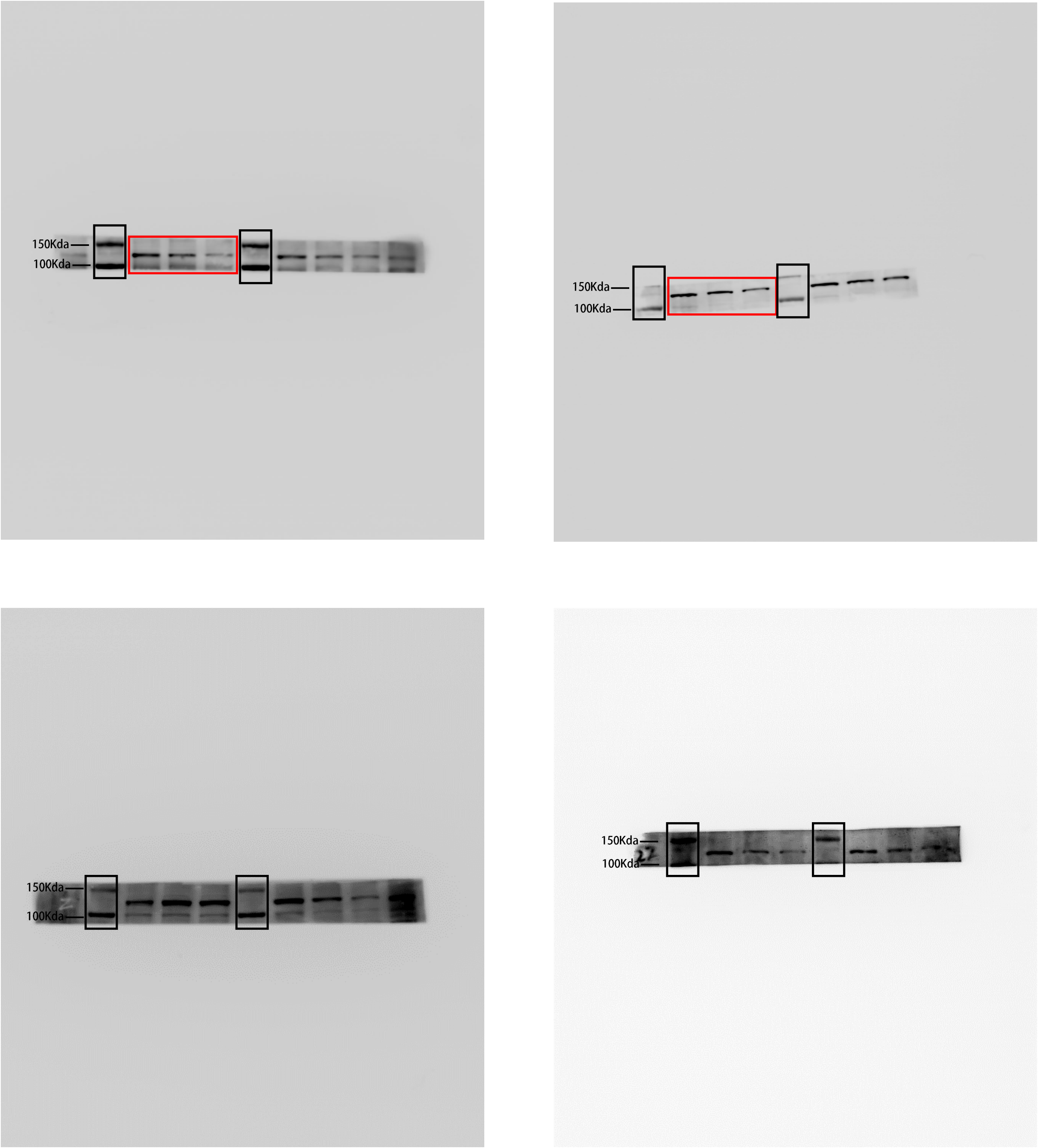

Original western blot image of ZEB1 shRNA, this band was cut in half for the other proteins before hybridization with the antibody.

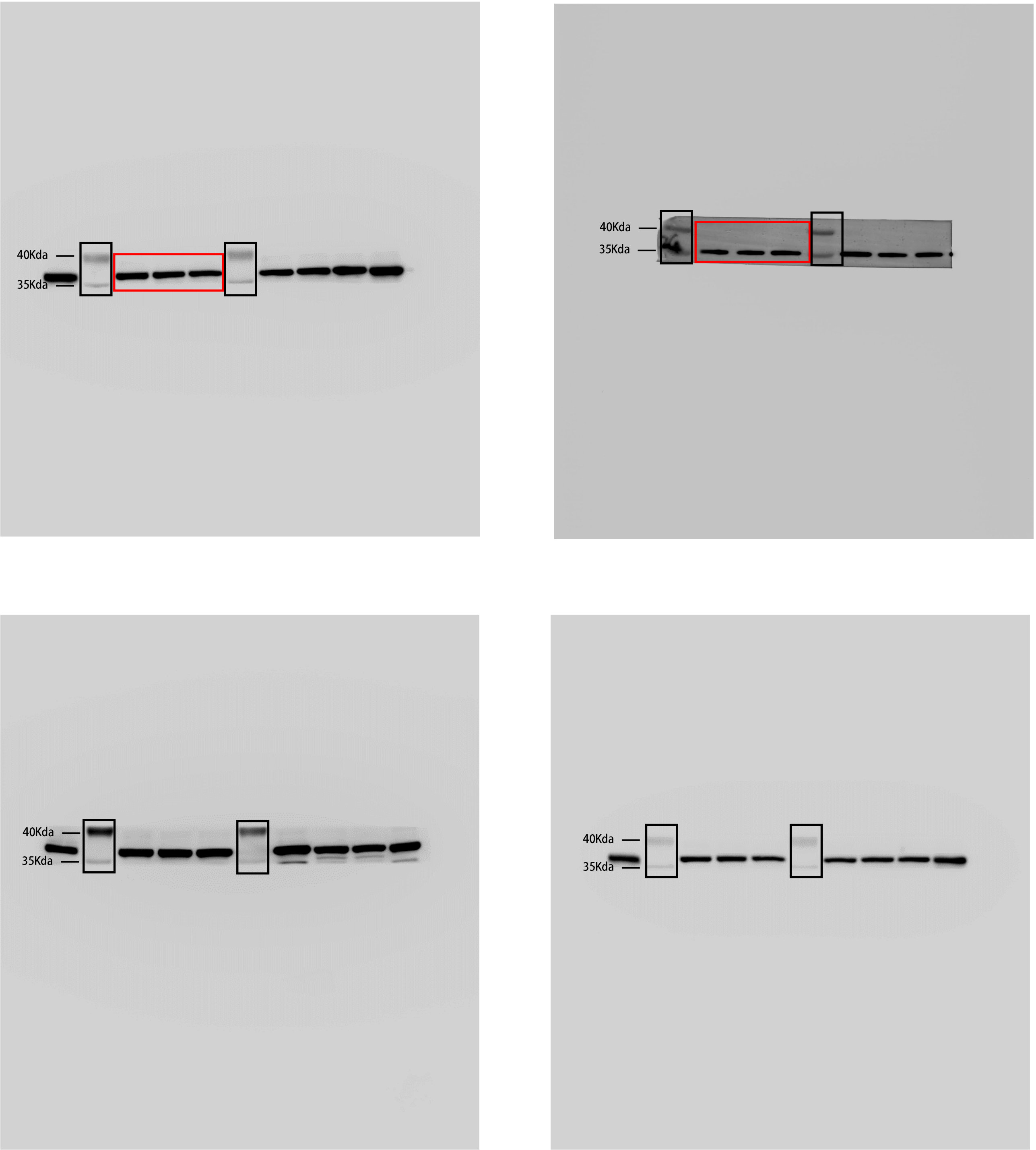

Original western blot image of GAPDH A549 (left) and H1299 (right).

Figure5 G

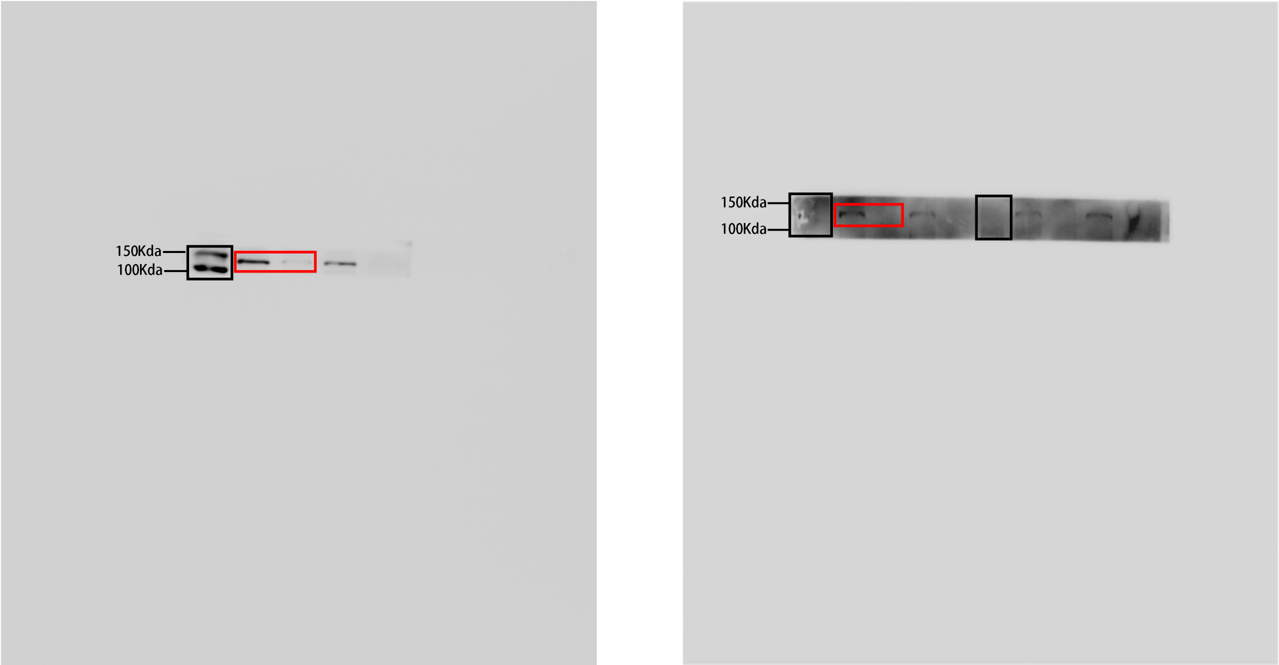

Original western blot image of ZEB1, this band was cut in half for the other proteins before hybridization with the antibody.

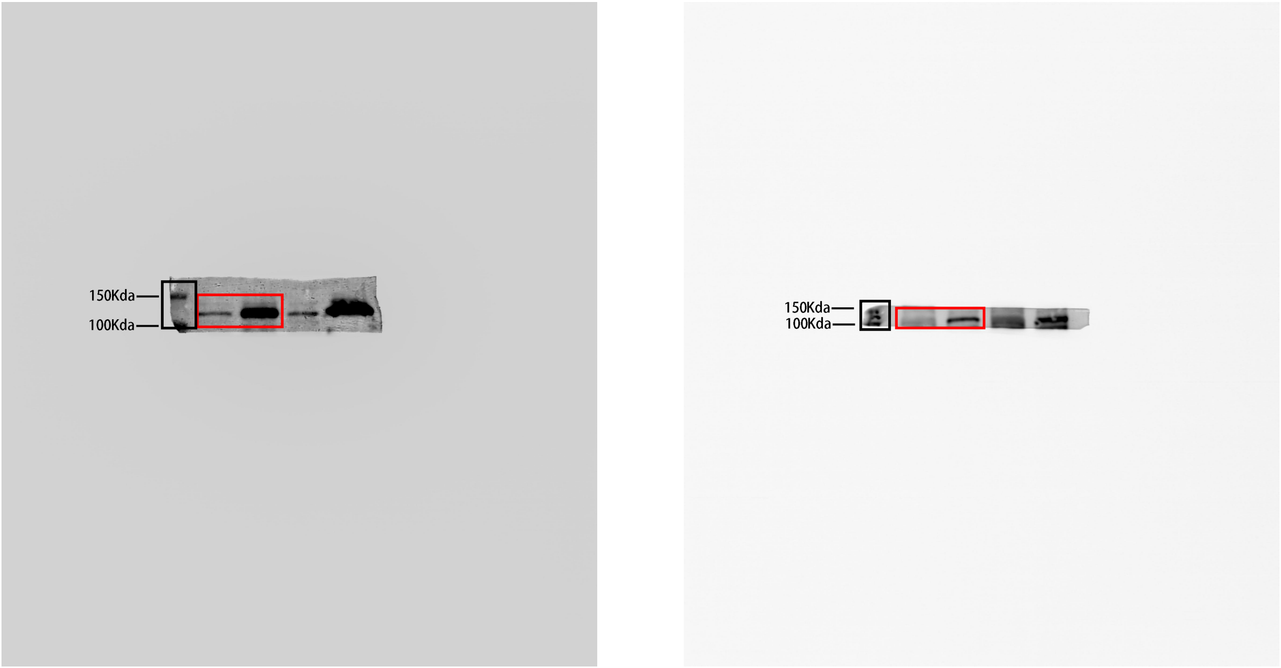

Original western blot image of E-cadherin in A549 (left) and H1299 (right), this band was cut in half for the other proteins before hybridization with the antibody.

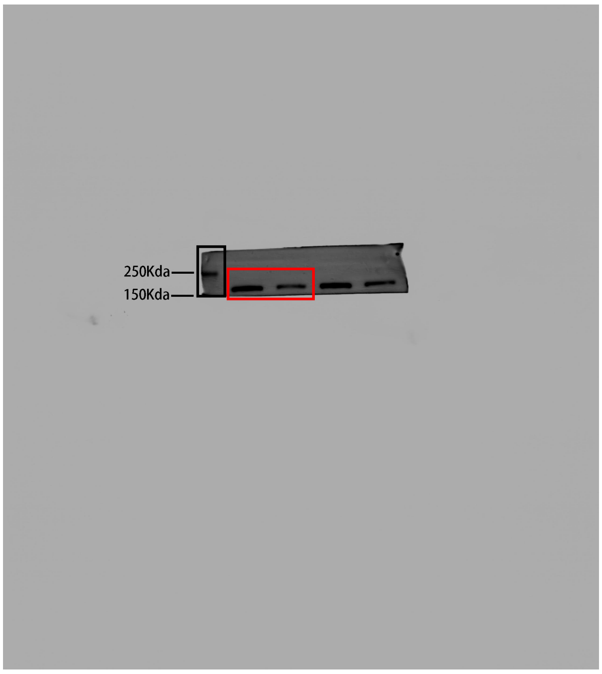

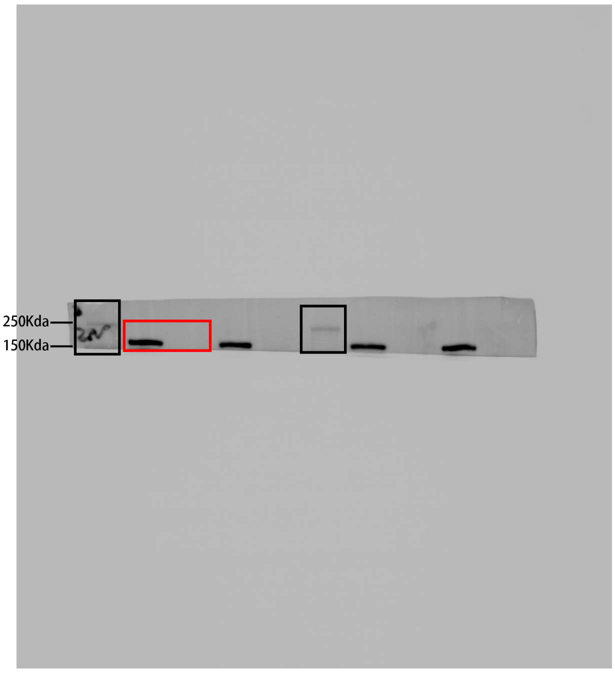

Original western blot image of N-cadherin in A549 (left) and H1299 (right), this band was cut in half for the other proteins before hybridization with the antibody.

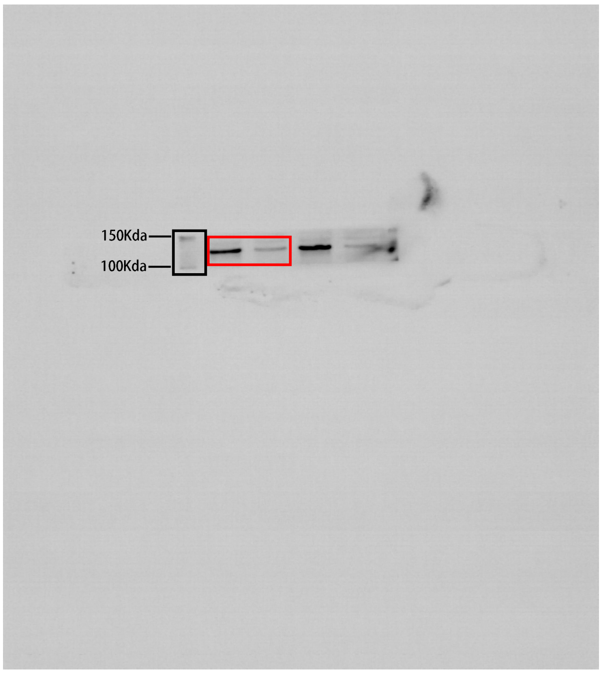

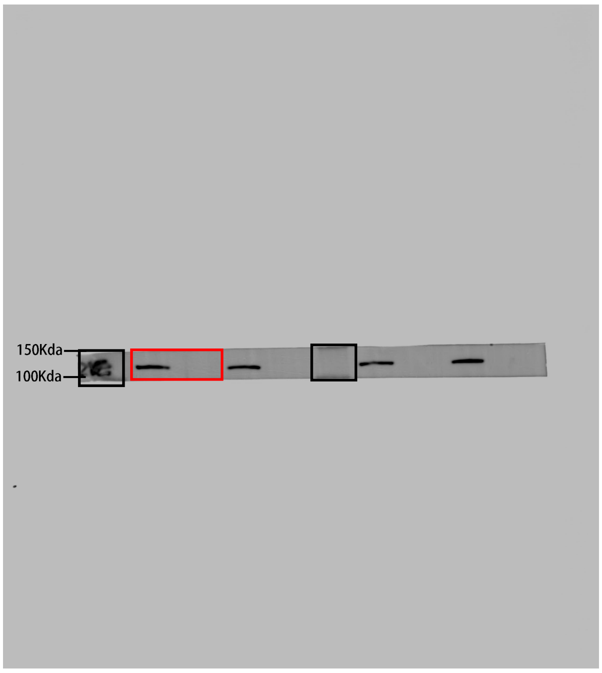

Original western blot image of VE-cadherin in A549 (left) and H1299 (right), this band was cut in half for the other proteins before hybridization with the antibody.

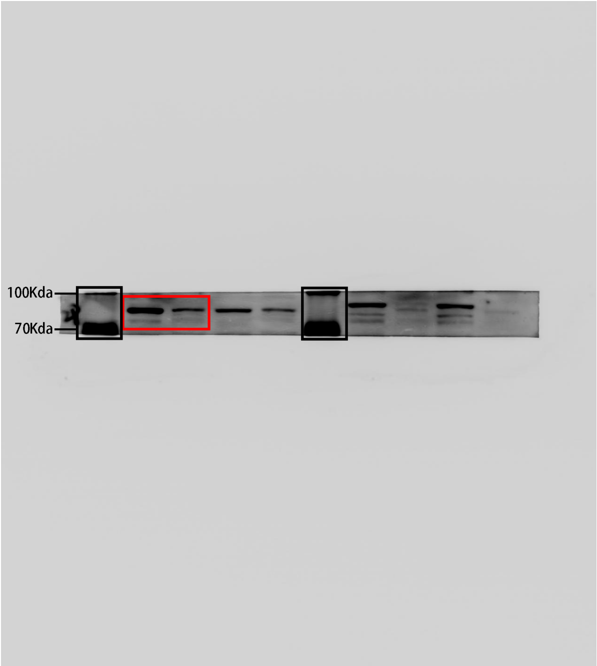

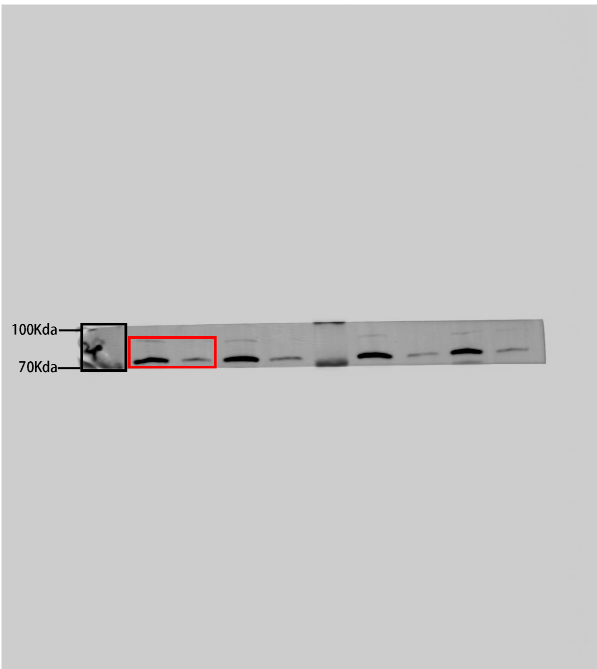

Original western blot image of MMP9 in A549 (left) and H1299 (right).

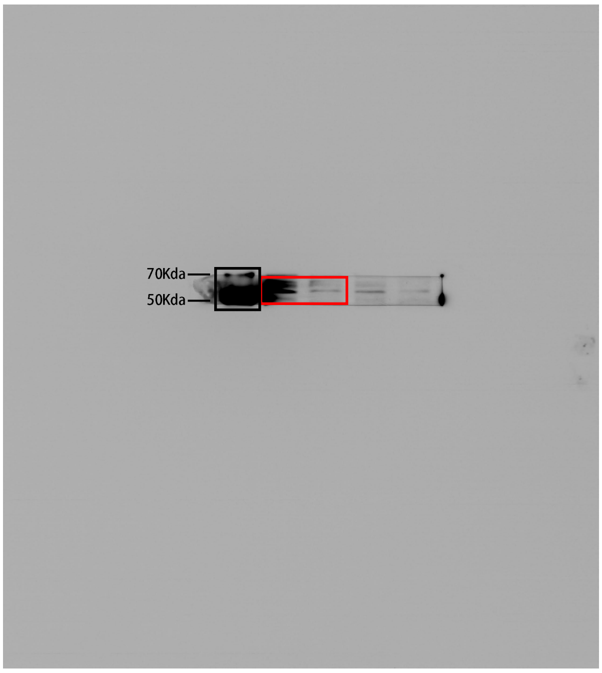

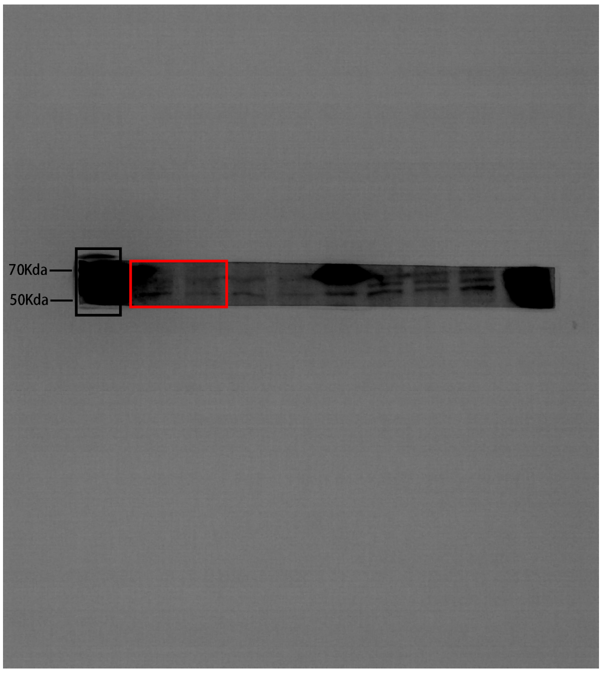

Original western blot image of MMP2 in A549 (left) and H1299 (right), this band was cut in half for the other proteins before hybridization with the antibody.

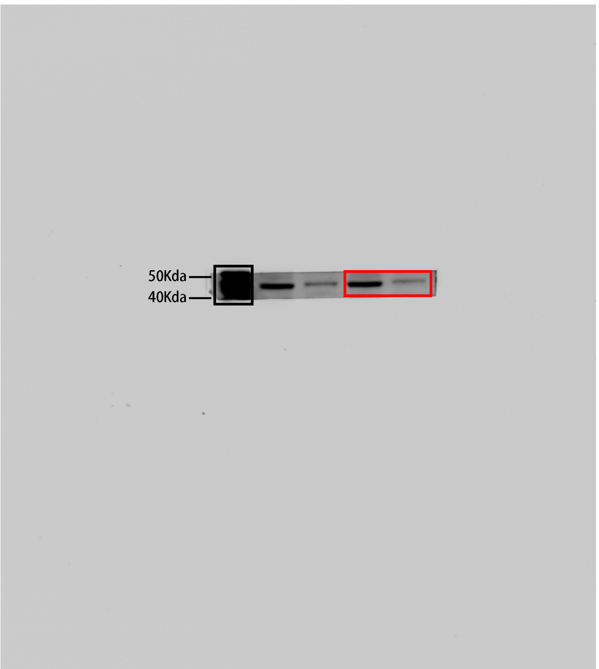

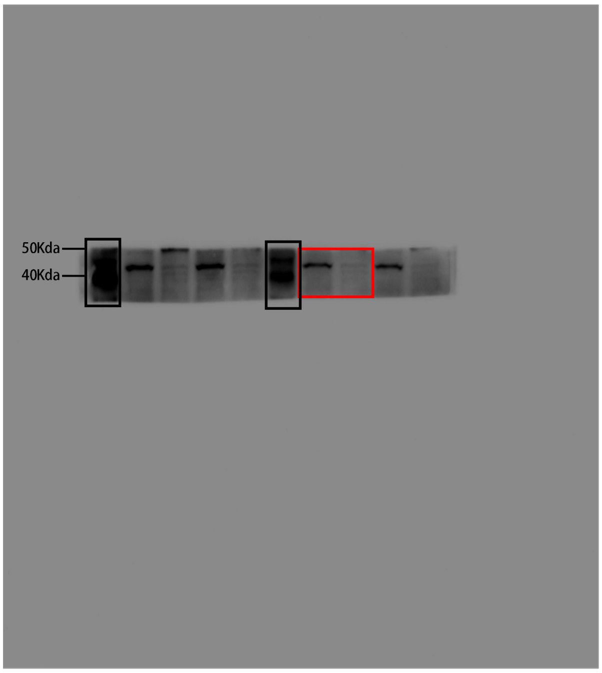

Original western blot image of VEGFA in A549 (left) and H1299 (right), this band was cut in half for the other proteins before hybridization with the antibody.

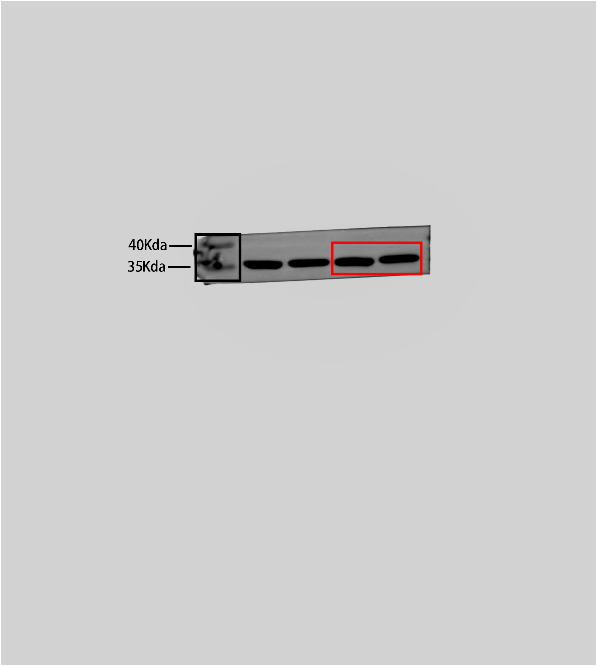

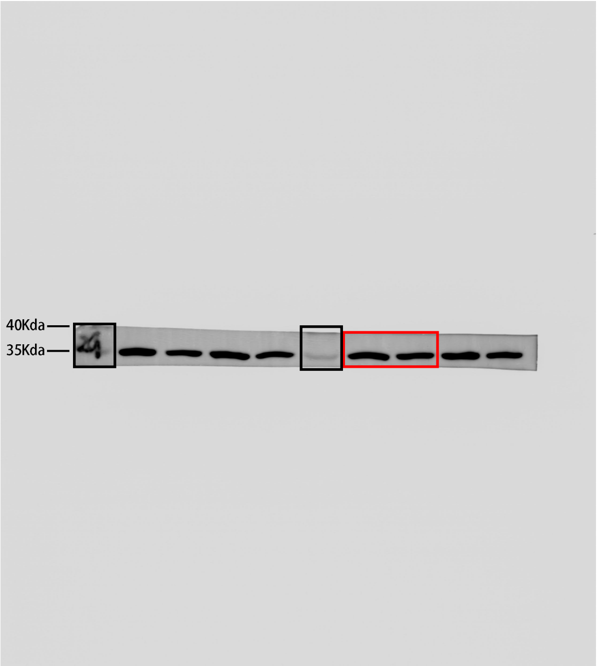

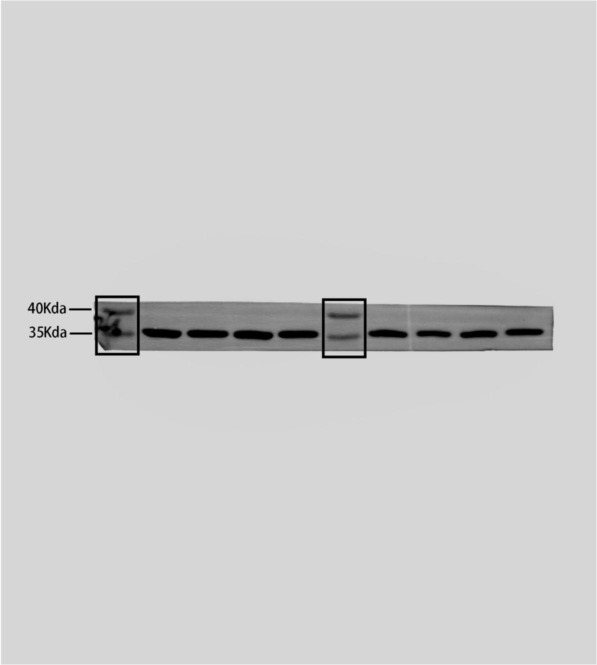

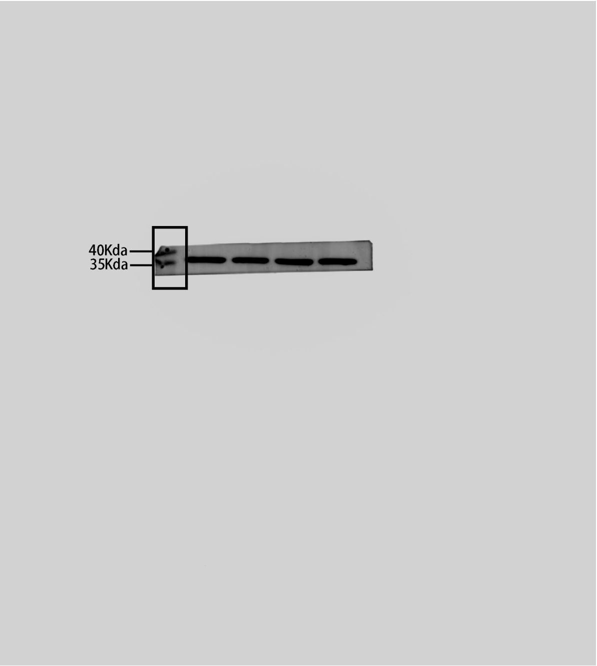

Original western blot image of GAPDH in A549 (left) and H1299 (right), this band was cut in half for the other proteins before hybridization with the antibody.

Note: the red box indicates the area of the original blot used in the main image; the black box indicates the hole where the protein mark is located. Most of the original strips provided were replicated three times in a single experiment. To facilitate antibody incubation targeting proteins from different regions, we trimmed the PVDF membrane before the primary antibody incubation. Shorter band lengths observed were attributed to the presence of proteins from different cells on both sides.
